# Supplementary material for: Retrospective evaluation of factors affecting successful fit testing of respiratory protective equipment during the early phase of COVID-19
Source: BMJ Open. 2023 May 25;13(5):e065068. doi: 10.1136/bmjopen-2022-065068 (PMC10230346; doi:10.1136/bmjopen-2022-065068)
Supplement: Supplementary data [file bmjopen-2022-065068supp001.pdf]

Appendix A

Learning from NHS England and NHS Improvement Quality Improvement Programme: FFP3 mask fit testing

A short toolkit for board members, infection prevention and control teams, managers and staff in all healthcare providers responsible for delivering an ongoing fit test training programme

Background to this toolkit

This short toolkit is designed to support NHS organisations to improve compliance with and uptake of FFP3 fit testing, improve staff experience, and enhance staff safety and well-being. It draws upon work undertaken by NHS England and NHS Improvement, and advice from other organisations, including the Health and Safety Executive, to identify best practice to support fit testing of FFP3 respirator masks and staff safety.

Summary of successful fit testing programmes

Using a rapid quality improvement approach, NHS England and NHS Improvement worked with 11 NHS organisations to identify the steps needed to ensure all staff required to wear an FFP3 mask are successfully fit tested with an appropriate mask. The improvement project enabled the participating organisations to rapidly identify barriers and implement solutions using small-scale tests of change. Through the course of the project several common themes emerged as being critical to the success of the fit testing process.

Collaboration

Departments key for facilitating successful fit testing include procurement, trust education and communication teams, health and safety teams, as well as staff networks, employee wellbeing, and occupational health teams. This ensures an integrated approach that coordinates supply, implementation, and education to deliver a programme with staff safety at its core.

Recommendation:

- Consider who is on the team, which other departments need to be involved and any additional support required for the delivery of the fit testing programme.

Education and communication with clinical and non-clinical staff

Evidence has suggested that some staff don't understand the difference between a fit check and a fit test. Several trusts identified gaps in understanding and embarked on staff education programmes which covered guidance on preparing for a fit test. This involved the use of posters, dedicated intranet pages, and regular bulletins. One trust set up a dedicated email account for advice about fit testing, and empowered fit test leads to have a proactive role in engaging departmental leads in understanding the importance of fit testing for staff. "PPE marshals" supported and trained staff in correct donning and doffing

procedures and monitoring adherence within the work setting following fit testing i.e. that fit checks were carried out and PPE was appropriately worn.

Staff experience was evaluated through the use of wellbeing charts, focus groups, surveys and analysis of staff journeys through the fit test process.

Recommendation:

- Ensure appropriate education and communication is in place for staff about the reasons for fit testing, and the process.

Accessibility of fit testing, including out of hours availability

Different sizes and types of organisations have different challenges in supporting staff to be able to get fit tested and this illustrated a need for a variety of approaches. Examples include the need to identify a risk-assessed and safe location (or locations for community organisations), increasing the frequency of fit test sessions, drop-in fit testing, a single port of call to arrange a fit test, and the use of a checklist. Some organisations trained redeployed staff as fit testers to expand the availability of testing, and introduced large-scale fit testing to more rapidly, fit-test large numbers of staff.

Recommendation: Ensure fit testing is widely available in accessible locations to maximise ability of staff to attend

Quality of fit testing

All organisations identified a need for robust training for fit testers including competency assessment and supervised sessions to ensure safe, high quality testing for staff. Checklists were common, and one organisation described the development of a video on how to correctly undertake a fit test. Clear local policies and guidelines based on HSE guidance, both for the fit test process and for staff, were developed to ensure consistency of approach.

Organisations used a mix of qualitative and quantitative testing; this project was not designed to detect the superiority of one method over the other, and did not do so. One trust reported that quantitative fit testing enabled multiple masks to be tested at one appointment, improving both success rates and efficiency of testing.

Other measures included training of fit testers by the mask supplier, inclusion of fit testing as part of organisational mandatory training, and ensuring a supply of a variety of types of mask from suppliers. Organisations identified that the availability of multiple masks for use in one fit test, was important in ensuring success, particularly for staff who had previously had an unsuccessful fit test.

Mask alternatives

This quality improvement programme focussed on FFP3 masks. Alternatives, for example, hoods and powered respirators, are available and were used where fitting an FFP3 mask

was not possible (on a case by case basis). It is important to recognise that not finding mask alternatives quickly, may put staff in a position of 'not wanting to let team down'.

HSE guidance at https://www.hse.gov.uk/respiratory-protective-equipment/fit-testing-basics.htm states that facial hair (stubble and beards) make it impossible to achieve a good seal of the respirator to the face. For staff wearing head coverings, tight fitting respirators such as FFP3 masks, the mask straps (for both fit testing and normal wear) do need to be worn in contact with the head and not the head covering. The reason for this is to lessen the chance of the straps slipping, which in turn could lead to a compromise in the face seal.

The FFP3 mask can be worn with the straps underneath the head covering. However, this should be handled sensitively - there must be a suitable environment for the wearer to don and doff the mask, and more importantly the head covering, in private.

Recommendation:

Further research into FFP3 masks and suitable alternatives, which are in line with HSE requirements, for staff who have beards or wear head coverings, is required.

Continuous measurement

Organisations highlighted the value of monitoring progress using continuous measurement - either using data collection resources provided by NHS England and NHS Improvement, or their own.

Cumulative analysis of the number of staff tested, total number of fit tests undertaken, and percentage of staff with a successful fit test enabled organisations to track progress with different types of FFP3 masks, and conduct further analysis to identify areas that required further input.

Recommendation:

- Staff experience should be discussed, with a broad range of staff representative of the demographic of staff employed by the trust, to enable mitigation of any issues quickly
- Link feedback from staff and testers, with other departments such as procurement (regarding changes in the availability of different types of masks)

Recording of results

All organisations identified the need for accurate and accessible recording of fit test outcomes, including negative results. Examples include a central database linked to health roster, registers, and certificates for staff. ESR has recently been updated to enable fit test results to be entered on staff records, which allows staff to download their results and notify other organisations if they work in more than one, or move posts. This should avoid unnecessary duplication of fit testing.

Recommendation:

- Record fit testing results at individual staff, departmental and trust level
- Continuously measure progress of fit testing using key benchmarks

- Ensure that all staff who are required to wear a FFP3 mask are regularly fit tested, and that there is organisational board level assurance

Redeployment

The availability of FFP3 mask alternatives such as hoods has reduced the need for redeployment. A redeployment algorithm developed by NHS England and NHS Improvement is available on the website.

https://www.england.nhs.uk/coronavirus/publication/supporting-fit-testing-steps-and-actions-to-be-taken-where-staff-may-require-the-use-of-ffp3-masks/

Recommendation:

- Ensure redeployment pathways are in place for staff who are not able to be successfully fit tested on available masks, or use alternatives.

Staff skin care

Once a member of staff has successfully been fit tested for a mask it is important to ensure that they take good care of their skin to prevent skin irritation and / or pressure ulcers. Guidance on how to help prevent facial skin damage beneath tight fitting face piece respirators (FFP 3 masks) is available.

https://www.england.nhs.uk/coronavirus/wp-content/uploads/sites/52/2020/03/C0129_Preventing_skin_damage_under_PPE_V6_29.10.20.pdf

Recommendations for fit testing

The structure of an organisation-wide fit testing programme should consider:

- **Compliance**
  - Governance structure must ensure that fit testing methodology meets with HSE standards
  - Include clear policies and guidelines
- **Fit testers**
  - Identify who they are, how many there are, the governance structure, competency assessment, their professional development and well-being
- **Staff to be fit tested**
  - Clarify the number of staff to be fit tested, their work areas, education about the fit test and support
- **Accessibility**
  - Consider the location of fit testing, number and timing of sessions, ease of booking of fit tests and the availability of urgent or short notice fit-testing
- **Equipment**
  - Quantitative and qualitative equipment required
  - Ensure consistent supply of FFP3 masks, ordering process and distribution, responsiveness to needs of the trust workforce
  - Ensure the availability of alternative PPE such as hoods

The following checklist was developed as part of the quality improvement programme.

The team is happy to share with resource with peers

Face Fit Testers
Checklist
FFP3 Disposable masks

NHS
Royal Free London
NHS Foundation Trust

**Fit Testers Name**		**Fit Test Method**	Qualitative
**Date**		**Assessors Name**	
**Work Location**			

	**Pass**	**Prompt**	**Fail**
**Understand where FFP3 masks must be worn[1]**			
Tester aware of Trust/PHE guidance on the correct PPE for task (i.e. AGP etc)			
**Can identify damage or defect in mask[2]**			
Check disposable mask for damage/defects			
**Practical - don mask and perform fit check**			
Demonstrates correct fitting of mask following IPC guidelines			
Demonstrates fit-check clearly, and instructs that it must be done every time mask is worn			
**Maintenance of testing equipment and room[3]**			
Check hood and pumps for damage/defects			
Checks nebuliser sprays			
Check room is suitable for fit test			
**Purpose fit testing and the fit test exercises**			
Can explain why we fit test staff and why we don't just do a static test			
**Limitations of qualitative fit testing[4]**			
Understands reasons to use machine test instead of taste test			
**Practical - perform a fit test**			
Checks for medical condition			
Explains test method clearly and purpose			
Sensitivity test carried out			
Understands option if user fails sensitivity test			
Ensures user performs fit check			
PPE worn if interferes with seal of RPE			
Carries out fit test exercises appropriately			
Can correct a failure in fit test (check[7])			
Only allows 1 retest on a mask model (check[7])			
Records results accurately			
Reminds retest period and reasons for retest			
**Doffing[12]**			
Can demonstrate how to safely doff and dispose of mask after use			

Caggiari S, et al. BMJ Open 2023; 13:e065068. doi: 10.1136/bmjopen-2022-065068

SCENARIO TEST			
	**Pass**	**Prompt**	**Fail**
**Understands problems that could prevent a successful fit test[8]**			
Identifies issues that might prevent person tasting solution			
Identifies issues with example images and suggests appropriate corrections			
**Recognise a poorly fitting FFP3 mask**			
Tester able to recognise poorly fitting mask (example images)			

[1] **Selection of adequate and suitable RPE.** Within healthcare setting the RPE is selected by IPC/PHE and fit testers would not be involved with this

[2] **Examination of RPE and the ability to identify poorly maintained facepieces** – this is for disposable only hence requirement to be able to check for defects. 3-5 example masks given with defects in some, such as missing valve, damaged seal etc.

[3] **Awareness of external factors that may affect the fit of the facepiece or the fit test result** – adjusted for QLFT rather than QNFT

[4] **The differences between, and the appropriate use of, QNFT and QLFT methods; capabilities and limitations of the fit test equipment** - adjusted to only reflect QLFT

[5] **Preparation of facepieces for fit testing** – REMOVED for QLFT

[6] **how to carry out diagnostic checks on the facepiece and the fit test equipment** – removed for QLFT

[7] When the first test is successful the knowledge should be checked by questioning

[8] Will give images of potential issues (holding a coffee, facial hair, headgear, PPE, straps worn incorrectly) tester to identify and explain how to correct

Facial Hair around the seal of the mask
Reminder to staff that they should be clean shaven when wearing RPE
Facial markings/piercing around the seal
Clothing or accessories (headscarf, glasses) that interfere with seal
Straps in wrong position

[9] **Interpretation of fit test results** – REMOVED for QLFT as the result is binary (reason for failure is already covered

[10] **Understanding of the differences between fit factor, workplace protection factor (WPF),† assigned protection factor and nominal protection factor (NPF)‡** - REMOVED N/A for NHS fit testers. RPE is decided at a national/hospital level, knowledge not required to be retained. Cover in training but this is not useful knowledge

[11] **HSE ACOPs and guidance that deal with fit testing of RPE.** - To be referred to in training, tested in the questions above.

[12] Not part of the HSE but felt it is necessary to add to NHS testing

Fit testing suggested reading

NHS England and NHS Improvement (2020) FFP3 respirators and other facial personal protective equipment (PPE). Available at: https://www.england.nhs.uk/coronavirus/secondary-care/infection-control/ppe/ffp3/ (Accessed 4 November 2020).

NHS England and NHS Improvement (2020) Fit testing algorithm. Available at: https://www.england.nhs.uk/coronavirus/publication/supporting-fit-testing-steps-and-actions-to-be-taken-where-staff-may-require-the-use-of-ffp3-masks/ (Accessed 4 November 2020).

NHS England and NHS Improvement (2020) Helping to prevent skin damage beneath tight fitting face respirators. Available at: https://www.england.nhs.uk/coronavirus/wp-content/uploads/sites/52/2020/03/C0129_Preventing_skin_damage_under_PPE_V6_29.10.20.pdf (Accessed 17 November 2020).

Public Health England, (2020a) Understanding the data: the impact of Covid 19 on BAME communities. Available at: https://assets.publishing.service.gov.uk/government/uploads/system/uploads/attachment_data/file/892376/COVID_stakeholder_engagement_synthesis_beyond_the_data.pdf [Accessed 19 October 2020]

Public Health England, (2020b) Disparities on the risk and outcomes of Covid 19. Available at: https://assets.publishing.service.gov.uk/government/uploads/system/uploads/attachment_data/file/908434/Disparities_in_the_risk_and_outcomes_of_COVID_August_2020_update.pdf_[Accessed 19 October 2020]

HSE guidance

HSE Guidance on respiratory protective equipment at work HSG53
https://www.hse.gov.uk/pubns/books/hsg53.htm

HSE Guidance on respiratory protective equipment (RPE) fit testing INDG479
https://www.hse.gov.uk/pubns/indg479.pdf

HSE website page on fit testing https://www.hse.gov.uk/respiratory-protective-equipment/fit-testing-basics.htm

HSE guidance on fit testing during COVID-19 https://www.hse.gov.uk/coronavirus/ppe-face-masks/face-mask-ppe-rpe.htm

British Industry Safety Federation Fit2fit companion guides https://www.bsif.co.uk/fit2fit-companions-released/
