# Supplementary material for: Retrospective evaluation of factors affecting successful fit testing of respiratory protective equipment during the early phase of COVID-19
Source: BMJ Open. 2023 May 25;13(5):e065068. doi: 10.1136/bmjopen-2022-065068 (PMC10230346; doi:10.1136/bmjopen-2022-065068)
Supplement: Supplementary data [file bmjopen-2022-065068supp002.pdf]

Appendix B

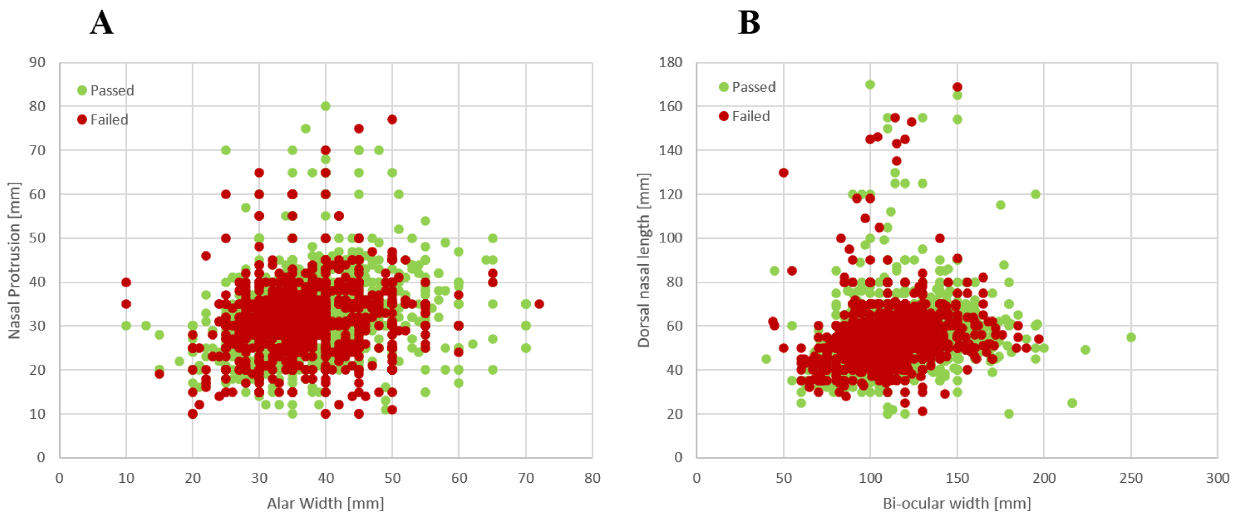

Categorical scatter plots of alar width [mm] versus nasal protrusion [mm] (A) and bi-ocular width [mm] versus dorsal nasal length [mm] (B) with respect to failed and passed fit tests at attempt 1, represented by red and green markers, respectively.
