# Supplementary material for: Retrospective evaluation of factors affecting successful fit testing of respiratory protective equipment during the early phase of COVID-19
Source: BMJ Open. 2023 May 25;13(5):e065068. doi: 10.1136/bmjopen-2022-065068 (PMC10230346; doi:10.1136/bmjopen-2022-065068)
Supplement: Author's manuscript [file bmjopen-2022-065068.draft_revisions.pdf]

BMJ Open

BMJ Open is committed to open peer review. As part of this commitment we make the peer review history of every article we publish publicly available.

When an article is published we post the peer reviewers' comments and the authors' responses online. We also post the versions of the paper that were used during peer review. These are the versions that the peer review comments apply to.

The versions of the paper that follow are the versions that were submitted during the peer review process. They are not the versions of record or the final published versions. They should not be cited or distributed as the published version of this manuscript.

BMJ Open is an open access journal and the full, final, typeset and author-corrected version of record of the manuscript is available on our site with no access controls, subscription charges or pay-per-view fees (http://bmjopen.bmj.com).

If you have any questions on BMJ Open's open peer review process please email info.bmjopen@bmj.com

BMJ Open

Retrospective analysis of a national fitting database for Respiratory Protective Equipment during the early phase of COVID-19.

Journal:	*BMJ Open*
Manuscript ID	bmjopen-2022-065068
Article Type:	Original research
Date Submitted by the Author:	12-Jul-2022

Complete List of Authors:	Caggiari, Silvia; University of Southampton, School of Health Sciences<br>Bader, Dan; University of Southampton, School of Health Sciences<br>Packman, Zoe; NHS England and NHS Improvement London<br>Robinson, Jane; NHS England and NHS Improvement London<br>Tranka , Sumeshni; NHS England and NHS Improvement London<br>Böhning, Dankmar ; University of Southampton, Southampton Statistical Sciences Research Institute<br>Worsley, Peter; University of Southampton

Keywords:	COVID-19, OCCUPATIONAL & INDUSTRIAL MEDICINE, Clinical audit < HEALTH SERVICES ADMINISTRATION & MANAGEMENT

SCHOLARONE™
Manuscripts

I, the Submitting Author has the right to grant and does grant on behalf of all authors of the Work (as defined in the below author licence), an exclusive licence and/or a non-exclusive licence for contributions from authors who are: i) UK Crown employees; ii) where BMJ has agreed a CC-BY licence shall apply, and/or iii) in accordance with the terms applicable for US Federal Government officers or employees acting as part of their official duties; on a worldwide, perpetual, irrevocable, royalty-free basis to BMJ Publishing Group Ltd ("BMJ") its licensees and where the relevant Journal is co-owned by BMJ to the co-owners of the Journal, to publish the Work in this journal and any other BMJ products and to exploit all rights, as set out in our licence.

The Submitting Author accepts and understands that any supply made under these terms is made by BMJ to the Submitting Author unless you are acting as an employee on behalf of your employer or a postgraduate student of an affiliated institution which is paying any applicable article publishing charge ("APC") for Open Access articles. Where the Submitting Author wishes to make the Work available on an Open Access basis (and intends to pay the relevant APC), the terms of reuse of such Open Access shall be governed by a Creative Commons licence – details of these licences and which Creative Commons licence will apply to this Work are set out in our licence referred to above.

Other than as permitted in any relevant BMJ Author's Self Archiving Policies, I confirm this Work has not been accepted for publication elsewhere, is not being considered for publication elsewhere and does not duplicate material already published. I confirm all authors consent to publication of this Work and authorise the granting of this licence.

For peer review only

BMJ Open

Title: Retrospective evaluation of factors affecting successful fit testing of Respiratory Protective Equipment during COVID-19

Authors: Silvia Caggiari [1], Dan L. Bader [1], Zoe Packman [2], Jane Robinson [2], Sue Tranka [2], Dankmar A. Böhning [3], Peter R. Worsley [1].

1. Clinical Academic Facility, School of Health Sciences, University of Southampton, Southampton, UK. SO17 1BJ
2. NHS England & Improvement, 3rd floor Skipton House, 80 London road, London, UK. SE1 6LH
3. Southampton Statistical Sciences Research Institute, University of Southampton, Southampton, UK, SO17 1BJ

Corresponding Author: Peter R Worsley. Clinical Academic Facility, School of Health Sciences, University of Southampton, Southampton, UK. SO17 1BJ

Email: P.R.Worsley@soton.ac.uk Tel: +44(0) 2381208287

Key Words: Personal Protective Equipment; COVID-19; Fitting; Occupational Health; Health Policy

Word Count: 3459

For peer review only

Abstract

Objectives: Respiratory protective equipment is critical to protect healthcare workers from COVID-19 infection, which includes Filtering Facepiece Respirators to prevent airborne transmission of the virus. There are reports of fitting issues within healthcare workers, although the factors affecting fitting outcomes are largely unknown. This study aimed to evaluate factors affecting respirator fitting outcomes within a national health service.

Methods: We conducted a secondary analysis of a national database of fit testing outcomes collated between July to August 2020. Key demographics including age, gender, ethnicity and face measurements were compared to fitting outcome in a variety of respirator designs. A mixed-effects logistic regression model was used to determine the factors which affected fit testing outcome.

Results: A total of 9,592 observations from 5,544 healthcare workers were included in the analysis. The mixed-effects logistic regression model identified that males experienced a significantly ($p<0.05$) higher fit test success than females (OR = 1.57; 95% CI: 1.31 to 1.88). Those with non-white ethnicities demonstrated significantly lower odds of successful mask fitting; Black (OR = 0.66; 95% CI: 0.51 to 0.86), Asian (OR = 0.60; 95% CI: 0.50 to 0.71) and Mixed (OR = 0.79; 95% CI: 0.64 to 0.99). Fitting success rate varied between mask type, with the lowest success rate of 49% and the highest of 94%.

Conclusion: During the early phase of COVID-19, females and non-White ethnicities were less likely to have a successful respirator mask fitting. Fitting success rates were variable between different makes and models of respirators, with more intelligent methods of fitting required to provide equal access to correctly fitting respirators.

For peer review only

Key messages

What is already known about this subject?

The literature suggested that there are differences in respirator fitting outcomes in healthcare workers, although to date, this has been limited to single centre studies and anecdotal evidence.

What are the new findings?

This is the first study to accommodate a national population of healthcare workers and evaluate fitting outcomes using several variations of N95/FFP3 respirators or equivalent designs. This study revealed that females experienced a significantly ($p<0.05$) lower fit test success than males and those with non-white ethnicities demonstrated significantly lower odds of successful mask fitting. Specific respirators demonstrated some bias in fitting outcomes, although further research is required to establish design factors in successful fitting outcomes.

How might it impact on clinical practice in the foreseeable future?

Respirator devices are critical to protect frontline staff working in COVID-19 environments. Healthcare settings require a range of masks to ensure successful fitting outcomes and improvements are required to provide more effective designs and fitting processes which are equitable for all individuals.

For peer review only

1. Introduction

The use of respiratory protective equipment (RPE) is vital in the prevention of airborne viral transmission for conditions such as influenza, SARS and SARS-CoV-2 (COVID-19). Indeed, a systematic review and meta-analysis demonstrated the use of N95 or FFP3respirators can reduce the risk of respiratory virus infection by 70%, suggesting mask use offers significant protection against the transmission of such respiratory viruses [1]. In the specific context of COVID-19, the risk of infection was significantly reduced amongst healthcare workers (HCWs) wearing Filtering Facepiece Respirators, highlighting the importance of RPE in the current pandemic [2]. As a result, there was an international effort to procure Filtering Facepiece Respirators, often termed N95 masks and FFP3 devices, creating an extreme demand on the associated supply chain [3 4].

Fit testing is used to minimise the inward leakage of the respirator when attached to the face and both qualitative fit tests (QLFT) and quantitative fit tests (QNFT) have been recommended [5 6]. However, respirators with sub-optimal fit are commonly used with a tightened strap used to create an airtight seal, thus endangering the health of the underlying skin on locations of the face [7]. Despite guidelines [8] and efforts to reduce skin damage, the prevalence of reported issues has remained high [9]. There are many factors which may influence successful RPE fitting [10]. These include the relationship between facial dimensions and RPE shape and the materials used within the mask which include both compliant and non-compliant designs [11]. Facial anthropometrics differences may influence the performance of RPE and there is already some evidence, albeit contradictory, that overall protection varies with gender-based differences in facial dimension [12-15].

In England, the National Health Service (NHS) has purchased a variety of FFP3 RPE devices to protect frontline HCWs in response to SARS-CoV-2 (COVID-19) as part of their pandemic preparedness stockpile. Recent studies in England has identified that male and White ethnicity healthcare workers are more likely to achieve RPE fit test success [16 17]. However, these were limited to a single healthcare centre limiting its generalisability. Thus, the present study aims to analyse this data retrospectively to answer the following key questions:

1. Do intrinsic factors, namely, gender identity, ethnic background, age and BMI affect the success rates of FFP3 mask fitting?
2. Is there a variation in performance of specific mask models in terms of fit test success over the number of attempts?
3. Do specific mask models match subject classification in terms of gender and ethnic group?
4. Do individual facial measurement parameters reflect the likelihood of successful mask fitting?

For peer review only

2. Methodology

This quality improvement study, involving National Health Service (NHS) hospitals in England, collated data regarding fit test outcomes in all healthcare workers. Each healthcare provider and individual participant were consented to take part in the study. Institutional ethics was approved to retrospectively analyse an anonymised version of the data set (ERGO-65166). Data was confidentially shared with the research group at the University of Southampton (UoS) and stored in accordance with the Data Protection Act 2018.

2.1 Fit testing data

Data included the type and make of RPE models used and success or failure of fitting according to two standardised and prescribed methods from the Health and Safety Executive, namely the qualitative fit test (QLFT) or the quantitative fit test (QNFT) [18 19]. This was conducted with a ToolKit provided (e-Appendix A) to each hospital trust, through which details of demographics were collected, including gender, ethnic background, age and BMI. In the latter case, BMIs were categories using the World Health Organization (WHO) classifications as underweight (<18.5 kg/m^2), optimal (18.5-25 kg/m^2), overweight (25-30 kg/m^2) and obese (>30 kg/m^2) [20]. In addition, five facial anthropometrical measurements were estimated with a paper-based ruler, namely, facial length, nasal length and protrusion, alar and bi-ocular width. Inclusion criteria consisted of hospital staff working in areas deemed necessary for RPE usage. Healthcare workers were initially fitted with a mask and assessed using the standardised tests. If they passed the mask was retained, and those that failed had further attempts with other mask models until successful (Figure 1). In some cases, HCWs were tested on multiple mask variants even when successful fitting was initially achieved, this was conducted to counter any supply chain issues with specific mask types.

[Insert Figure 1 near here]

Data collection was carried out in a 5 week period between July and August 2020. ToolKits were electronically distributed to a number of hospitals in England, identified with an anonymised code and responsible to name an experienced fit tester for the recruitment of staff members. All data were electronically collected, anonymised and stored securely within NHS England and Improvement.

2.2 Data analysis

Data detailed the result of up to five fit test attempts for FFP3 respirator masks in a cohort of >5000 hospital staff. These data were reviewed to ensure consistency in the analytical methods. This involved a cleansing process to minimise redundancy and remove outliers (from the paper-ruler facial measurements), missing or undeclared data. Subsequent review of the data included:

For peer review only

- Ethnic backgrounds initially categorized into 24 groups and subsequently into four major ethnic backgrounds, namely, White, Asian, Black and Mixed. Further analysis was conducted with respect to sub-groups within each ethnicities associated with the specific country of origin.
- A few individuals identified their gender as "Non binary", representing only 0.1% of the total cohort. These were excluded from the analysis, as they precluded an analysis of the effect of gender on success rates of mask fitting.

2.3 Statistical Analysis

Cleaned data was imported into Stata (Stata 16.0, StataCorp LLC, USA), where a basic model assessment was performed using the Bayesian information criterion (BIC). The model with the lowest BIC value was selected incorporating the mask model, gender, ethnicity and age. Following this evaluation, a mixed-effects logistic regression model was used to determine the ORs and their 95% confidence intervals (95% CI) for factors which affected fit testing outcome using a collated data set across each of the five fit test attempts. Analysis was conducted while adjusting for multiple risk factors and potential confounders. This approach accounted for nested data (as several, different masks were fitted to the same person) and for within-subject correlation by means of a random effect for subject.

Explorative data analysis was also conducted to evaluate trends between fitting outcomes within different ethnicities and gender and BMI categories. In addition, a cumulative frequency approach was used to investigate the distribution of the facial measurements. Cluster analyses were conducted to identify any trends in the measurements associated with fit test outcome.

3. Results

3.1 Overview of the data

A total of 9,592 observations from 5,512 healthcare workers were included in the regression model (132 data entries were removed from the quality control process). This included data from 47 NHS Hospitals in England, where healthcare workers were fit tested with various mask designs up to five times. Most of the individuals, approximately 78% of the total for all attempts, were aged between 24 and 54 years of age. Those that were younger (18-24 years) represented 9% of the cohort, whereas those in the older categories (up to 74 years of age) represented 13% of the participants.

The number of attempts differed between individuals, with a steady decline from attempt 1 to 5. To review briefly, there were 5512 (Table 1), 2191, 1072, 560 and 257, in the first, second, third, fourth and fifth accumulative attempts, respectively. There was a small increase in the proportion of Asian

For peer review only

individuals between the first (20.3%) and fifth (27.2%) attempts, while the gender and BMI categories remained largely unchanged. The vast majority of the individuals (~80% of the total for all attempts) were identified to be in the middle two BMI classifications. Individuals with a BMI under 18 kg/m^2 represented the smallest proportion of the cohort (3-5%) in all five attempts.

Table 1: Summary of anthropometric data of participants at the first fit test attempt.

Gender	*Percentage of healthcare workers fit tested (First Attempt)*
Female	79.6%
Male	20.4%
White (Caucasian)	64.3%
Asian/Asian British	20.3%
Black/African/Caribbean	8.9%
Mixed/Multiple backgrounds	6.5%
Under 18.5 kg/m^2	3.3%
18.5-25 kg/m^2	50.2%
25-30 kg/m^2	31.7%
Over 30 kg/m^2	14.9%

3.2 Mixed-effects logistic regression model

The final model after adjusting for covariates in our multilevel mixed-effects logistic regression analysis indicated that the odds of experiencing a successful FFP3 mask fitting were significantly ($p<0.05$) higher among those who were male (OR = 1.57; 95% CI: 1.31 to 1.88) compared to females. In addition, the youngest age category (18-24 years) had a significantly lower odds of success fitting compared to all older age categories (OR=2.55-3.45, $p<0.01$). Those with non-white ethnicities demonstrated lower odds of successful mask fitting, with black (OR = 0.66; 95% CI: 0.51 to 0.86), Asian (OR = 0.60; 95% CI: 0.50 to 0.71) and mixed (OR = 0.79; 95% CI: 0.64 to 0.99) ethnic groups all being statistically significantly ($p<0.05$) when compared with the white ethnic group (Table 2).

For peer review only

Table 2: Odds ratios (OR) of a successful FFP3 mask fit test outcome and the corresponding 95% confidence intervals for each factor adjusted for all other factors using multilevel mixed-effects logistic regression.

Factor		Odds Ratio	~~Std. Err.~~	z	p> |z|	95% Confidence Interval
Mask Model	3M1863			*1 (base)*		
	3M1863+	.933	~~0.0914~~	-0.71	0.476	0.769 to 1.130
	3M 1873V	1.529	~~0.209~~	3.11	**0.002**	1.170 to 1.997
	3M8833	1.534	~~0.156~~	4.20	**0.001**	1.256 to 1.873
	Cardinal RFP3FV	0.513	~~0.060~~	-5.75	**0.001**	0.408 to 0.6438
	FSM18	0.806	~~0.141~~	-1.23	0.217	0.573 to 1.135
	3M1873	0.893	~~0.130~~	-0.77	0.439	0.671 to 1.189
	Other*	1.013	~~0.088~~	0.15	0.883	0.855 to 1.200
Ethnicity	White			*1 (base)*		
	Black	0.661	~~0.087~~	-3.15	**0.002**	0.511 to 0.855
	Asian	0.597	~~0.052~~	-5.92	**0.001**	0.503 to 0.708
	Mixed	0.792	~~0.089~~	-2.06	**0.039**	0.635 to 0.988
Sex	Female			*1 (base)*		
	Male	1.571	~~0.145~~	4.89	**0.001**	1.311 to 1.883
Age	18-24 years			*1 (base)*		
	25-34 years	3.410	~~0.340~~	10.47	**0.001**	2.710 to 4.291
	35-44 years	2.55	~~0.312~~	7.67	**0.001**	2.010 to 3.247
	45-54 years	2.750	~~0.339~~	8.21	**0.001**	2.160 to 3.501
	55+ years	3.457	~~0.504~~	8.50	**0.001**	2.598 to 4.602

LR test vs. logistic model: chibar2(01) = 524.33 Prob >= chibar2 = 0.0001

The likelihood ratio test (last line Table 2) showed that the random effect for subject is strongly required and the simple logistic regression approach would have been not sufficient here. In addition, analysis revealed that there were no significant interactions of mask with sex, age, ethnicity.

For peer review only

3.3 Explorative Data Analysis

With respect to ethnicity and gender, the group of White male participants yielded the highest success rates, with absolute differences of ≥ 4% compared to all other groups (Table 3). In a similar manner, the White females yielded a higher success rate than their corresponding ethnic comparators, with a difference of ≥ 5 %. Asian and Mixed background ethnic groups yielded the lowest success rate of 60.3% and 60.4%, respectively. Closer examination of the fit test outcome also revealed the lowest success rate of approximately 56% associated with Bangladeshi HCWs, although they only represent a small proportion (54/2133, ~2.5%) of the corresponding Asian cohort.

For peer review only

Table 3: The absolute differences in global fit test success rate with reference to gender and ethnicity. Created by the authors

Gender and Ethnic Group (participants, % success rate)	White Male	White Female	Asian/Asian British Male	Asian/Asian British Female	Black/African/ Caribbean Male	Black/African/ Caribbean Female	Mixed/Multiple backgrounds Male	Mixed/Multiple backgrounds Female
White Male (1046, 74.3%)	-	-5.8	-4.4	-14.0	-3.5	-10.8	-7.6	-13.9
White Female (4988, 68.5%)		-	1.4	-8.2	2.3	-5.0	-1.8	-8.1
Asian/Asian British Male (627, 69.9%)				-9.6	0.9	-6.4	-3.2	-9.5
Asian/Asian British Female (1506, 60.3%)				-	10.5	3.2	6.4	0.1
Black/African/Caribbean Male (168, 70.8%)					-	-7.3	-4.1	-10.4
Black/African/Caribbean Female (642, 63.5%)						-	3.2	-3.1
Mixed/Multiple backgrounds Male (168, 66.7%)							-	-6.3
Mixed/Multiple backgrounds Female (447, 60.4%)								-

For peer review only

3.5 Fit test outcomes according to facial measurements

Facial measurements were examined with respect to the total cohort of 5544 HCWs. The cumulative frequency trends for each of the facial parameters were found to be normally distributed and, as a result, parametric descriptors were used for analysis. Close examination of the data suggests that there was a limited discrimination between facial measurements and fit test outcomes, with a wide range of values attributed to individuals who both passed and failed at each attempt (e-Appendix B).

4. Discussion

A multi-centre quality improvement study was implemented to explore the factors associated with fitting outcomes for Filtering Facepiece Respirators (FFP3) during the early phase of COVID-19 (July and August 2020). Conducted through NHS England and Improvement, data describing the fitting outcomes from multiple mask models was completed for over 5000 HCWs. The multivariate analysis revealed that women yielded a significantly lower odds ratio of fit test success rates compared to males. In addition, Asian, Black and Mixed ethnic backgrounds individuals had a lower fit test success rate than White ethnicities, with White males yielding the highest success rate (74%) and Asian and Mixed ethnicities females the lowest (60%) (Table 3). It was also noted that individuals with low BMI i.e. < 18.5 kg/m^2 many of whom were Asian, yielded a very low fit test success rate.

Gender-based differences have been associated with RPE fit testing outcomes in a number of studies, the majority of which demonstrate that female participants yield a significantly lower RPE success rate, and as a result need a range of mask models for successful fitting [12-16 21-27]. This is in contrast to a recent study in the England, where no gender differences were identified [15], although, where data were accessible, it did highlight gender differences according to mask type. To date, many studies have not made gender-based comparisons of RPE performance for individual mask models, restricting gender comparison to overall fit testing success rates. The present study has observed that specific mask models are an independent indicator of RPE fitting success. Indeed, increasing RPE choice could improve user success rates and reduce gender-based differences, with one previous study demonstrating that the inclusion of two additional mask models accounted for a 20% improvement in female pass rates [28]. Subsequently, several studies recommend a variety of RPE should be made available to ensure successful fit testing [23 29-36].

The present study observed clear ethnicity-based differences in RPE outcomes, both for males and females. Small comparative studies have demonstrated lower pass rates for Black and Asian females [14 16 31 37]. In addition, studies of Asian populations have consistently yielded higher rates of fit-test

For peer review only

failure within Chinese, Koreans, Taiwanese and Iranian cohorts, further emphasising the importance of considering facial dimensions of the relevant population in RPE design [13 21 22 24-26 34-36 38 39]. These differences are likely to have resulted from the known effects of ethnicity on anthropometric facial features [40]. Therefore, the RPE models currently available do not provide comparable protection in a multi-ethnic workforce, thus disadvantaging those from minority groups. This implies that RPE designs evaluated during this study did not accommodate the heterogeneity in facial features across diverse user populations due to the limited nature of the in panels used for international standards e.g., EN-149. It is also of note that fit testing success varied across the different BMI ranges, with the underweight category (<18.5kg/m^2) yielding the highest failure rates. Closer analysis of the present findings also revealed that 40% of individuals with low BMI were Asian, 52% of whom did not achieve success at any attempt at fit testing. This suggests that both ethnicity related anthropometrics of face shape and body mass index are contributing factors in fit test outcomes. Indeed, the current respirator fit panels do not accommodate those in the low BMI range, further evidence has shown that fitting will alter dependent on changes in an individual's weight [41]. Indeed, the soft tissue composition of the face could effect a change in the contact between the mask and the underlying skin, with more bony prominences creating a less conforming surface from which masks can create a seal.

4.1 Limitations

This secondary analysis of quality improvement data was limited by the nature in which individual hospital trust collected and documented fit testing. Indeed, a small proportion of data was omitted during the data cleaning process in order to conduct robust analysis. This was performed using defined criteria and enabled robust analysis, for example removing miss-labelled data or entries which did not include key parameters e.g. mask type or ethnicity. The results of this retrospective analysis are only reflective of the time period of investigation (July-August 2020), with improvements to mask designs and fitting processes evident during the latter stages of the pandemic. The data presented in the present study is also a product of the international demands on PPE supply chain [3], with a diverse range of masks included in the data set. As a result, RPE in different English hospitals was variable and procurement dependent. In addition, implementing a comprehensive fit-testing program is a financial and logistical challenge [42], limiting the feasibility to test all HCWs on different FFP3 mask models. These changes would be partly determined by the availability of the mask models, which was likely to vary between healthcare centres and where new models have been introduced into the market during the study period. Any comparisons between respirators should be viewed with caution, there was no random allocation of mask types across the fit testing attempts. Since not all participants fit-tested all respirator types, and those selected for subsequent attempts were not randomised, this introduced a selection bias which precludes meaningful inference about the fit success rate of different respirator designs. The anthropometric data was limited by the use of paper-based rulers,

For peer review only

with limited accuracy and reliability. Given the findings of gender and ethnicity differences, further evaluation of anthropometrics using accurate scanning technologies are required.

4.2 Clinical Implications

In practice, poorly fitted RPE designs impede both functional capacity and user safety [43 44]. Widespread concerns in areas of RPE fit-test access, availability and training have been highlighted during the pandemic [45 46]. Notably, for HCWs using RPE for prolonged periods, skin damage has been reported with a variable prevalence of between 42% and 97%. This has been attributed to ill-fitting RPE and may account for higher rates of adverse reactions within Ethnic minorities individuals [9 47-49]. In many countries there have been significant improvements in the supply chain of RPE devices and a range of models is available to many healthcare institutions. Further research into the design and fitting of RPE must consider the demographics of the healthcare workforce including the diverse range of ethnicities, ages and genders. Design panels incorporating established data sets of different sub-groups of demographics could inform new designs and international standards (e.g. ISO/DIS 16976-6) by which FFP3 masks can be manufactured. Healthcare institutions should maintain a range of RPE devices to accommodate successful fitting for their diverse workforce and data shared with industrial representatives to ensure the available designs are fit for purpose.

5. Conclusion

This secondary analysis of respirator fit testing outcomes revealed distinct trends in fit testing outcomes related to both gender and ethnicity. Indeed, white males were the most likely to yield a successful fit test outcome and ethnic minority females the least likely. In many cases, repeat fit testing with different respirator models was required to ensure individual staff had a correctly fitting device. Further research is needed to provide improvements in the fit testing approach resulting in a more intelligent selection process.

Acknowledgements

We would like to thank all the participating hospital trusts support data collection during the Quality Improvement Initiative. This study was commissioned by NHS England and Improvement. The secondary analysis of data was supported by UK Research and Innovation (UKRI) as part of a funded project “A Bio-Engineering approach for the SAFE design and fitting of Respiratory Protective Equipment (BE-SAFE RPE)” (Ref EP/V045563/1).

For peer review only

Competing interest

There are no conflicts of interest for any of the authors in this manuscript.

Patient and Public Involvement

Patients and public (healthcare workers) were consulted on the challenges associated with getting correctly fitting respirator masks and this formed the motivation for the quality improvement study. These were consulted regarding data collection forms and analysis.

References

1. Liang M, Gao L, Cheng C, et al. Efficacy of face mask in preventing respiratory virus transmission: A systematic review and meta-analysis. *Travel Med Infect Dis* 2020;36:101751. doi: https://doi.org/10.1016/j.tmaid.2020.101751 [published Online First: 2020/05/31]
2. Li Y, Liang M, Gao L, et al. Face masks to prevent transmission of COVID-19: A systematic review and meta-analysis. *Am J Infect Control* 2020 doi: https://doi.org/10.1016/j.ajic.2020.12.007 [published Online First: 2020/12/22]
3. Feinmann J. PPE: what now for the global supply chain? *BMJ* 2020;369:m1910. doi: https://doi.org/10.1136/bmj.m1910
4. Miller FA, Young SB, Dobrow M, et al. Vulnerability of the medical product supply chain: the wake-up call of COVID-19. *BMJ Qual Saf* 2021;30(4):331-35. doi: https://doi.org/10.1136/bmjqs-2020-012133 [published Online First: 2020/11/04]
5. NIOSH. RCT-APR-STP-0068: Total Inward Leakage Test for Half-mask Air-purifying Particulate Respirators. *42 CFR Part 84, Subpart K: National Institute for Occupational Safety and Health* 2009
6. OSHA. 1910.134: Respiratory protection. App A: Fit testing procedures (mandatory). Subpart I: Personal Protective Equipment: Occupational Safety and Health Administration.
7. Worsley PR, Spratt F, Bader DL. COVID19: Challenging tissue viability in both patients and clinicians. *Journal of Tissue Viability* 2020;29(3):153-54. doi: https://doi.org/10.1016/j.jtv.2020.06.003
8. NHS England and NHS Improvement. Helping prevent facial skin damage beneath personal protective equipment. 2019
9. Abiakam N, Worsley P, Jayabal H, et al. Personal protective equipment related skin reactions in healthcare professionals during COVID-19. *Int Wound J* 2021;18(3):312-22. doi: https://doi.org/10.1111/iwj.13534 [published Online First: 2021/01/29]

For peer review only

10. Regli A, Sommerfield A, von Ungern-Sternberg BS. The role of fit testing N95/FFP2/FFP3 masks: a narrative review. *Anaesthesia* 2021;76(1):91-100. doi: https://doi.org/10.1111/anae.15261

11. Lawrence RB, Duling MG, Calvert CA, et al. Comparison of performance of three different types of respiratory protection devices. *J Occup Environ Hyg* 2006;3(9):465-74. doi: https://doi.org/10.1080/15459620600829211 [published Online First: 2006/07/22]

12. Oestenstad RK, Perkins LL. An assessment of critical anthropometric dimensions for predicting the fit of a half-mask respirator. *American Industrial Hygiene Association Journal* 1992;53(10):639-44. doi: https://doi.org/10.1080/15298669291360283

13. Han D-H, Choi K-L. Facial Dimensions and Predictors of Fit for Half-Mask Respirators in Koreans. *AIHA Journal* 2003;64(6):815-22. doi: https://doi.org/10.1080/15428110308984877

14. Manganyi J, Wilson KS, Rees D. Quantitative Respirator Fit, Face Sizes, and Determinants of Fit in South African Diagnostic Laboratory Respirator Users. *Ann Work Expo Health* 2017;61(9):1154-62. doi: https://doi.org/10.1093/annweh/wxx077 [published Online First: 2017/11/15]

15. Green S, Gani A, Bailey M, et al. Fit-testing of respiratory protective equipment in the UK during the initial response to the COVID-19 pandemic. *Journal of Hospital Infection* 2021;113:180-86. doi: https://doi.org/10.1016/j.jhin.2021.04.024

16. Carvalho CYM, Schumacher J, Greig PR, et al. Prospective observational study of gender and ethnicity biases in respiratory protective equipment for healthcare workers in the COVID-19 pandemic. *BMJ Open* 2021;11(5):e047716. doi: 10.1136/bmjopen-2020-047716

17. Ascott A, Crowest P, de Sausmarez E, et al. Respiratory personal protective equipment for healthcare workers: impact of sex differences on respirator fit test results. *Br J Anaesth* 2021;126(1):e48-e49. doi: 10.1016/j.bja.2020.10.016 [published Online First: 2020/11/14]

18. Brienza D, Vallely J, Karg P, et al. An MRI investigation of the effects of user anatomy and wheelchair cushion type on tissue deformation. *Journal of Tissue Viability* 2018;27(1):42-53. doi: https://doi.org/10.1016/j.jtv.2017.04.001

19. Bogie KM, Nuseibeh I, Bader DL. Early progressive changes in tissue viability in the seated spinal cord injured subject. *Paraplegia* 1995;33:141-47.

20. Bakhsh TM. Results of unicompartmental knee replacement. *Saudi Medical Journal* 2007;28(7):1062-64.

21. Han DH. Fit factors for quarter masks and facial size categories. *The Annals of Occupational Hygiene* 2000;44(3):227-34. doi: https://doi.org/10.1016/S0003-4878(99)00087-3

22. Kim H, Han DH, Roh YM, et al. Facial anthropometric dimensions of Koreans and their associations with fit of quarter-mask respirators. *Ind Health* 2003;41(1):8-18. doi: https://doi.org/10.2486/indhealth.41.8 [published Online First: 2003/04/04]

For peer review only

23. McMahon E, Wada K, Dufresne A. Implementing fit testing for N95 filtering facepiece respirators: Practical information from a large cohort of hospital workers. *American Journal of Infection Control* 2008;36(4):298-300. doi: https://doi.org/10.1016/j.ajic.2007.10.014

24. Yu Y, Jiang L, Zhuang Z, et al. Fitting Characteristics of N95 Filtering-Facepiece Respirators Used Widely in China. *PLOS ONE* 2014;9(1):e85299. doi: https://doi.org/10.1371/journal.pone.0085299

25. Honarbakhsh M, Jahangiri M, Ghaem H, et al. Qualitative Fit Testing of Medium - Size N95/FFP2 Respirators on Iranian Health Care Workers. *Health Scope* 2018;7(4):e62884. doi: https://doi.org/10.5812/jhealthscope.62884 [published Online First: 2018-05-29]

26. Huh YJ, Jeong HM, Lim J, et al. Fit Characteristics of N95 Filtering Facepiece Respirators and the Accuracy of the User Seal Check among Koreans. *Infect Control Hosp Epidemiol* 2018;39(1):104-07. doi: https://doi.org/10.1017/ice.2017.271 [published Online First: 2018/01/19]

27. Oestenstad RK, Dillion HK, Perkins LL. Distribution of Faceseal Leak Sites on a Half-Mask Respirator and Their Association with Facial Dimensions. *American Industrial Hygiene Association Journal* 1990;51(5):285-90. doi: https://doi.org/10.1080/15298669091369664

28. Zhuang Z, Coffey CC, Ann RB. The effect of subject characteristics and respirator features on respirator fit. *J Occup Environ Hyg* 2005;2(12):641-9. doi: https://doi.org/10.1080/15459620500391668 [published Online First: 2005/11/22]

29. Ciotti C, Pellissier G, Rabaud C, et al. Effectiveness of respirator masks for healthcare workers, in France. *Med Mal Infect* 2012;42(6):264-9. doi: https://doi.org/10.1016/j.medmal.2012.05.001 [published Online First: 2012/06/12]

30. Winter S, Thomas JH, Stephens DP, et al. Particulate face masks for protection against airborne pathogens - one size does not fit all: an observational study. *Crit Care Resusc* 2010;12(1):24-7. [published Online First: 2010/03/04]

31. Spies A, Wilson KS, Ferrie R. Respirator fit of a medium mask on a group of South Africans: a cross-sectional study. *Environ Health* 2011;10:17. doi: https://doi.org/10.1186/1476-069x-10-17 [published Online First: 2011/03/17]

32. Earle-Richardson G, Fiske T, Wyckoff S, et al. Respiratory fit testing for farmworkers in the Black Dirt region of Hudson Valley, New York. *J Agromedicine* 2014;19(4):346-53. doi: https://doi.org/10.1080/1059924x.2014.945711 [published Online First: 2014/10/03]

33. Kim H, Baek JE, Seo HK, et al. Assessing real-time performances of N95 respirators for health care workers by simulated workplace protection factors. *Ind Health* 2015;53(6):553-61. doi: https://doi.org/10.2486/indhealth.2014-0259 [published Online First: 2015/09/01]

34. Foereland S, Robertsen O, Hegseth MN. Do Various Respirator Models Fit the Workers in the Norwegian Smelting Industry? *Saf Health Work* 2019;10(3):370-76. doi: https://doi.org/10.1016/j.shaw.2019.06.004 [published Online First: 2019/09/10]

For peer review only

For peer review only - http://bmjopen.bmj.com/site/about/guidelines.xhtml

35. Fakherpour A, Jahangiri M, Seif M, et al. Quantitative fit testing of filtering face-piece respirators during the COVID-19 pandemic reveals anthropometric deficits in most respirators available in Iran. *Journal of Environmental Health Science and Engineering* 2021;19(1):805-17. doi: https://doi.org/10.1007/s40201-021-00648-3

36. Fakherpour A, Jahangiri M, Seif M. Qualitative fitting characteristics of filtering face-piece respirators on Iranian people. *J Environ Health Sci Eng* 2020;18(2):587-97. doi: https://doi.org/10.1007/s40201-020-00484-x

37. Brazile WJ, Buchan RM, Sandfort DR, et al. Respirator Fit and Facial Dimensions of Two Minority Groups. *Applied Occupational and Environmental Hygiene* 1998;13(4):233-37. doi: https://doi.org/10.1080/1047322X.1998.10390073

38. Lin YC, Chen CP. Characterization of small-to-medium head-and-face dimensions for developing respirator fit test panels and evaluating fit of filtering facepiece respirators with different faceseal design. *PLoS One* 2017;12(11):e0188638. doi: https://doi.org/10.1371/journal.pone.0188638 [published Online First: 2017/11/28]

39. Zhang X, Jia N, Wang Z. The relationship between the filtering facepiece respirator fit and the facial anthropometric dimensions among Chinese people. *Ind Health* 2020;58(4):318-24. doi: https://doi.org/10.2486/indhealth.2019-0158 [published Online First: 2019/12/04]

40. Zhuang Z, Landsittel D, Benson S, et al. Facial anthropometric differences among gender, ethnicity, and age groups. *Ann Occup Hyg* 2010;54(4):391-402. doi: https://doi.org/10.1093/annhyg/meq007 [published Online First: 2010/03/12]

41. Roberge R, Zhuang Z, Stein L. Association of body mass index with facial dimensions for defining respiratory fit panels. American Industrial Hygiene Conference and Exposition. Chicago, Illinois, 2006.

42. Wilkinson IJ, Pisaniello D, Ahmad J, et al. Evaluation of a large-scale quantitative respirator-fit testing program for healthcare workers: survey results. *Infect Control Hosp Epidemiol* 2010;31(9):918-25. doi: https://doi.org/110.1086/655460 [published Online First: 2010/07/28]

43. Sathasivam S, Walker PS. The conflicting requirements of laxity and conformity in total knee replacement. *J Biomech* 1999;32(3):239-47. doi: https://doi.org/10.1016/s0021-9290(98)00139-0 [published Online First: 1999/03/27]

44. Fidler H. PPE: 'one size fits all' design is a fallacy that's putting female health staff at risk. . *Nursing Standard* 2020

45. RCN. Second Personal Protective Equipment Survey of UK Nursing Staff Report: Use and availability of PPE during the COVID-19 pandemic. 2020

46. Gilroy R. PPE designed for rugby players not BME women, warns MP. *Nursing Times* 2020

47. Zhao Y, Liang W, Luo Y, et al. Personal protective equipment protecting healthcare workers in the Chinese epicentre of COVID-19. *Clin Microbiol Infect* 2020;26(12):1716-18. doi: https://doi.org/10.1016/j.cmi.2020.07.029 [published Online First: 2020/07/28]

For peer review only

500 48. Jiang Q, Song S, Zhou J, et al. The Prevalence, Characteristics, and Prevention Status of Skin Injury Caused by Personal Protective Equipment Among Medical Staff in Fighting COVID-19: A Multicenter, Cross-Sectional Study. *Adv Wound Care (New Rochelle)* 2020;9(7):357-64. doi: https://doi.org/10.1089/wound.2020.1212 [published Online First: 2020/04/23]

504 49. Lan J, Song Z, Miao X, et al. Skin damage among health care workers managing coronavirus disease-2019. *J Am Acad Dermatol* 2020;82(5):1215-16. doi: https://doi.org/10.1016/j.jaad.2020.03.014 [published Online First: 2020/03/17]

507

For peer review only

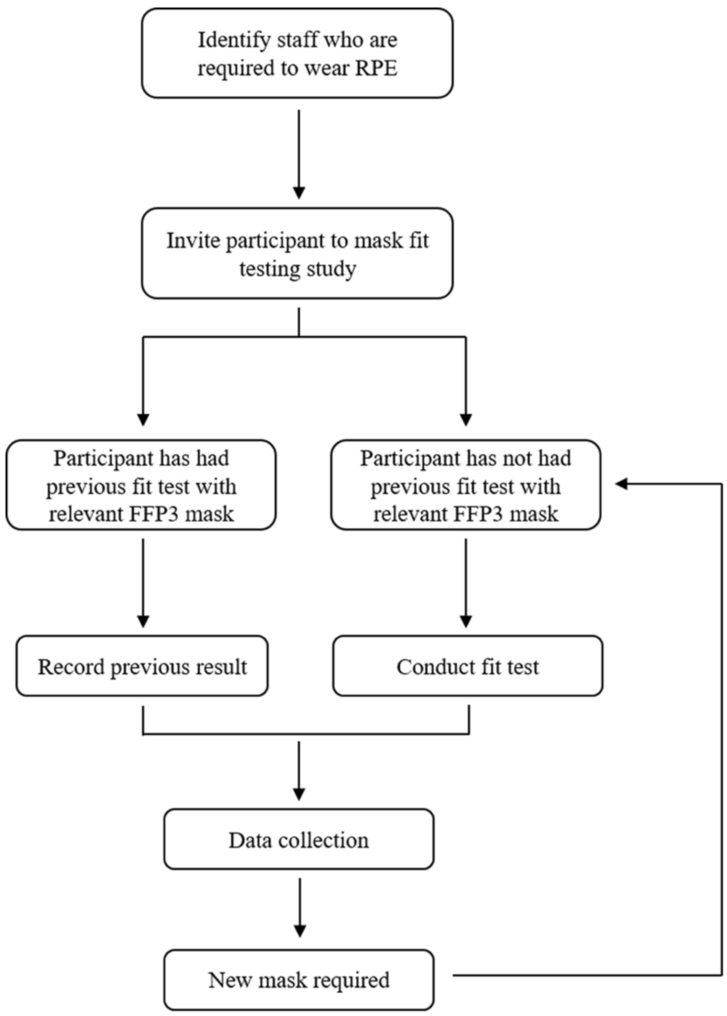

Figure 1: Schematic of mask fitting and data collection process.

1322x1869mm (96 x 96 DPI)

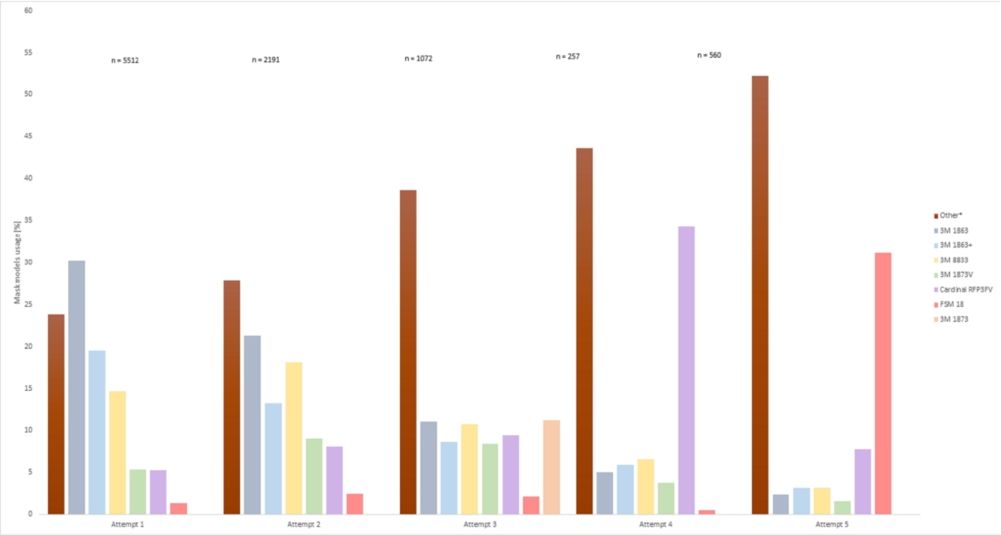

Figure 2: The percentage of mask type used for each fitting attempt.

1852x1057mm (96 x 96 DPI)

Learning from NHS England and NHS Improvement Quality Improvement Programme: FFP3 mask fit testing

A short toolkit for board members, infection prevention and control teams, managers and staff in all healthcare providers responsible for delivering an ongoing fit test training programme

Background to this toolkit

This short toolkit is designed to support NHS organisations to improve compliance with and uptake of FFP3 fit testing, improve staff experience, and enhance staff safety and well-being. It draws upon work undertaken by NHS England and NHS Improvement, and advice from other organisations, including the Health and Safety Executive, to identify best practice to support fit testing of FFP3 respirator masks and staff safety.

Summary of successful fit testing programmes

Using a rapid quality improvement approach, NHS England and NHS Improvement worked with 11 NHS organisations to identify the steps needed to ensure all staff required to wear an FFP3 mask are successfully fit tested with an appropriate mask. The improvement project enabled the participating organisations to rapidly identify barriers and implement solutions using small-scale tests of change. Through the course of the project several common themes emerged as being critical to the success of the fit testing process.

Collaboration

Departments key for facilitating successful fit testing include procurement, trust education and communication teams, health and safety teams, as well as staff networks, employee wellbeing, and occupational health teams. This ensures an integrated approach that coordinates supply, implementation, and education to deliver a programme with staff safety at its core.

Recommendation:

- Consider who is on the team, which other departments need to be involved and any additional support required for the delivery of the fit testing programme.

Education and communication with clinical and non-clinical staff

Evidence has suggested that some staff don't understand the difference between a fit check and a fit test. Several trusts identified gaps in understanding and embarked on staff education programmes which covered guidance on preparing for a fit test. This involved the use of posters, dedicated intranet pages, and regular bulletins. One trust set up a dedicated email account for advice about fit testing, and empowered fit test leads to have a proactive role in engaging departmental leads in understanding the importance of fit testing for staff. "PPE marshals" supported and trained staff in correct donning and doffing

For peer review only

procedures and monitoring adherence within the work setting following fit testing i.e. that fit checks were carried out and PPE was appropriately worn.

Staff experience was evaluated through the use of wellbeing charts, focus groups, surveys and analysis of staff journeys through the fit test process.

Recommendation:

- Ensure appropriate education and communication is in place for staff about the reasons for fit testing, and the process.

Accessibility of fit testing, including out of hours availability

Different sizes and types of organisations have different challenges in supporting staff to be able to get fit tested and this illustrated a need for a variety of approaches. Examples include the need to identify a risk-assessed and safe location (or locations for community organisations), increasing the frequency of fit test sessions, drop-in fit testing, a single port of call to arrange a fit test, and the use of a checklist. Some organisations trained redeployed staff as fit testers to expand the availability of testing, and introduced large-scale fit testing to more rapidly, fit-test large numbers of staff.

Recommendation: Ensure fit testing is widely available in accessible locations to maximise ability of staff to attend

Quality of fit testing

All organisations identified a need for robust training for fit testers including competency assessment and supervised sessions to ensure safe, high quality testing for staff. Checklists were common, and one organisation described the development of a video on how to correctly undertake a fit test. Clear local policies and guidelines based on HSE guidance, both for the fit test process and for staff, were developed to ensure consistency of approach.

Organisations used a mix of qualitative and quantitative testing; this project was not designed to detect the superiority of one method over the other, and did not do so. One trust reported that quantitative fit testing enabled multiple masks to be tested at one appointment, improving both success rates and efficiency of testing.

Other measures included training of fit testers by the mask supplier, inclusion of fit testing as part of organisational mandatory training, and ensuring a supply of a variety of types of mask from suppliers. Organisations identified that the availability of multiple masks for use in one fit test, was important in ensuring success, particularly for staff who had previously had an unsuccessful fit test.

Mask alternatives

This quality improvement programme focussed on FFP3 masks. Alternatives, for example, hoods and powered respirators, are available and were used where fitting an FFP3 mask

For peer review only

was not possible (on a case by case basis). It is important to recognise that not finding mask alternatives quickly, may put staff in a position of 'not wanting to let team down'.

HSE guidance at https://www.hse.gov.uk/respiratory-protective-equipment/fit-testing-basics.htm states that facial hair (stubble and beards) make it impossible to achieve a good seal of the respirator to the face. For staff wearing head coverings, tight fitting respirators such as FFP3 masks, the mask straps (for both fit testing and normal wear) do need to be worn in contact with the head and not the head covering. The reason for this is to lessen the chance of the straps slipping, which in turn could lead to a compromise in the face seal.

The FFP3 mask can be worn with the straps underneath the head covering. However, this should be handled sensitively - there must be a suitable environment for the wearer to don and doff the mask, and more importantly the head covering, in private.

Recommendation:

Further research into FFP3 masks and suitable alternatives, which are in line with HSE requirements, for staff who have beards or wear head coverings, is required.

Continuous measurement

Organisations highlighted the value of monitoring progress using continuous measurement - either using data collection resources provided by NHS England and NHS Improvement, or their own.

Cumulative analysis of the number of staff tested, total number of fit tests undertaken, and percentage of staff with a successful fit test enabled organisations to track progress with different types of FFP3 masks, and conduct further analysis to identify areas that required further input.

Recommendation:

- Staff experience should be discussed, with a broad range of staff representative of the demographic of staff employed by the trust, to enable mitigation of any issues quickly
- Link feedback from staff and testers, with other departments such as procurement (regarding changes in the availability of different types of masks)

Recording of results

All organisations identified the need for accurate and accessible recording of fit test outcomes, including negative results. Examples include a central database linked to health roster, registers, and certificates for staff. ESR has recently been updated to enable fit test results to be entered on staff records, which allows staff to download their results and notify other organisations if they work in more than one, or move posts. This should avoid unnecessary duplication of fit testing.

Recommendation:

- Record fit testing results at individual staff, departmental and trust level
- Continuously measure progress of fit testing using key benchmarks

For peer review only - http://bmjopen.bmj.com/site/about/guidelines.xhtml

- Ensure that all staff who are required to wear a FFP3 mask are regularly fit tested, and that there is organisational board level assurance

Redeployment

The availability of FFP3 mask alternatives such as hoods has reduced the need for redeployment. A redeployment algorithm developed by NHS England and NHS Improvement is available on the website.

https://www.england.nhs.uk/coronavirus/publication/supporting-fit-testing-steps-and-actions-to-be-taken-where-staff-may-require-the-use-of-ffp3-masks/

Recommendation:

- Ensure redeployment pathways are in place for staff who are not able to be successfully fit tested on available masks, or use alternatives.

Staff skin care

Once a member of staff has successfully been fit tested for a mask it is important to ensure that they take good care of their skin to prevent skin irritation and / or pressure ulcers. Guidance on how to help prevent facial skin damage beneath tight fitting face piece respirators (FFP 3 masks) is available.

https://www.england.nhs.uk/coronavirus/wp-content/uploads/sites/52/2020/03/C0129_Preventing_skin_damage_under_PPE_V6_29.10.20.pdf

Recommendations for fit testing

The structure of an organisation-wide fit testing programme should consider:

- **Compliance**
  - Governance structure must ensure that fit testing methodology meets with HSE standards
  - Include clear policies and guidelines
- **Fit testers**
  - Identify who they are, how many there are, the governance structure, competency assessment, their professional development and well-being
- **Staff to be fit tested**
  - Clarify the number of staff to be fit tested, their work areas, education about the fit test and support
- **Accessibility**
  - Consider the location of fit testing, number and timing of sessions, ease of booking of fit tests and the availability of urgent or short notice fit-testing
- **Equipment**
  - Quantitative and qualitative equipment required
  - Ensure consistent supply of FFP3 masks, ordering process and distribution, responsiveness to needs of the trust workforce
  - Ensure the availability of alternative PPE such as hoods

For peer review only - http://bmjopen.bmj.com/site/about/guidelines.xhtml

The following checklist was developed as part of the quality improvement programme.

The team is happy to share with resource with peers

Face Fit Testers
Checklist
FFP3 Disposable masks

NHS
Royal Free London
NHS Foundation Trust

**Fit Testers Name**		**Fit Test Method**	Qualitative
**Date**		**Assessors Name**	
**Work Location**			

	**Pass**	**Prompt**	**Fail**
**Understand where FFP3 masks must be worn[1]**			
Tester aware of Trust/PHE guidance on the correct PPE for task (i.e. AGP etc)			
**Can identify damage or defect in mask[2]**			
Check disposable mask for damage/defects			
**Practical - don mask and perform fit check**			
Demonstrates correct fitting of mask following IPC guidelines			
Demonstrates fit-check clearly, and instructs that it must be done every time mask is worn			
**Maintenance of testing equipment and room[3]**			
Check hood and pumps for damage/defects			
Checks nebuliser sprays			
Check room is suitable for fit test			
**Purpose fit testing and the fit test exercises**			
Can explain why we fit test staff and why we don't just do a static test			
**Limitations of qualitative fit testing[4]**			
Understands reasons to use machine test instead of taste test			
**Practical - perform a fit test**			
Checks for medical condition			
Explains test method clearly and purpose			
Sensitivity test carried out			
Understands option if user fails sensitivity test			
Ensures user performs fit check			
PPE worn if interferes with seal of RPE			
Carries out fit test exercises appropriately			
Can correct a failure in fit test (check[7])			
Only allows 1 retest on a mask model (check[7])			
Records results accurately			
Reminds retest period and reasons for retest			
**Doffing[12]**			
Can demonstrate how to safely doff and dispose of mask after use			

For peer review only

For peer review only - http://bmjopen.bmj.com/site/about/guidelines.xhtml

SCENARIO TEST			
	**Pass**	**Prompt**	**Fail**
**Understands problems that could prevent a successful fit test[8]**			
Identifies issues that might prevent person tasting solution			
Identifies issues with example images and suggests appropriate corrections			
**Recognise a poorly fitting FFP3 mask**			
Tester able to recognise poorly fitting mask (example images)			

[1] **Selection of adequate and suitable RPE.** Within healthcare setting the RPE is selected by IPC/PHE and fit testers would not be involved with this

[2] **Examination of RPE and the ability to identify poorly maintained facepieces** – this is for disposable only hence requirement to be able to check for defects. 3-5 example masks given with defects in some, such as missing valve, damaged seal etc.

[3] **Awareness of external factors that may affect the fit of the facepiece or the fit test result** – adjusted for QLFT rather than QNFT

[4] **The differences between, and the appropriate use of, QNFT and QLFT methods; capabilities and limitations of the fit test equipment** - adjusted to only reflect QLFT

[5] **Preparation of facepieces for fit testing** – REMOVED for QLFT

[6] **how to carry out diagnostic checks on the facepiece and the fit test equipment** – removed for QLFT

[7] When the first test is successful the knowledge should be checked by questioning

[8] Will give images of potential issues (holding a coffee, facial hair, headgear, PPE, straps worn incorrectly) tester to identify and explain how to correct

Facial Hair around the seal of the mask
Reminder to staff that they should be clean shaven when wearing RPE
Facial markings/piercing around the seal
Clothing or accessories (headscarf, glasses) that interfere with seal
Straps in wrong position

[9] **Interpretation of fit test results** – REMOVED for QLFT as the result is binary (reason for failure is already covered

[10] **Understanding of the differences between fit factor, workplace protection factor (WPF),† assigned protection factor and nominal protection factor (NPF)‡** - REMOVED N/A for NHS fit testers. RPE is decided at a national/hospital level, knowledge not required to be retained. Cover in training but this is not useful knowledge

[11] **HSE ACOPs and guidance that deal with fit testing of RPE.** - To be referred to in training, tested in the questions above.

[12] Not part of the HSE but felt it is necessary to add to NHS testing

For peer review only - http://bmjopen.bmj.com/site/about/guidelines.xhtml

Fit testing suggested reading

NHS England and NHS Improvement (2020) FFP3 respirators and other facial personal protective equipment (PPE). Available at: https://www.england.nhs.uk/coronavirus/secondary-care/infection-control/ppe/ffp3/ (Accessed 4 November 2020).

NHS England and NHS Improvement (2020) Fit testing algorithm. Available at: https://www.england.nhs.uk/coronavirus/publication/supporting-fit-testing-steps-and-actions-to-be-taken-where-staff-may-require-the-use-of-ffp3-masks/ (Accessed 4 November 2020).

NHS England and NHS Improvement (2020) Helping to prevent skin damage beneath tight fitting face respirators. Available at: https://www.england.nhs.uk/coronavirus/wp-content/uploads/sites/52/2020/03/C0129_Preventing_skin_damage_under_PPE_V6_29.10.20.pdf (Accessed 17 November 2020).

Public Health England, (2020a) Understanding the data: the impact of Covid 19 on BAME communities. Available at: https://assets.publishing.service.gov.uk/government/uploads/system/uploads/attachment_data/file/892376/COVID_stakeholder_engagement_synthesis_beyond_the_data.pdf [Accessed 19 October 2020]

Public Health England, (2020b) Disparities on the risk and outcomes of Covid 19. Available at: https://assets.publishing.service.gov.uk/government/uploads/system/uploads/attachment_data/file/908434/Disparities_in_the_risk_and_outcomes_of_COVID_August_2020_update.pdf_[Accessed 19 October 2020]

HSE guidance

HSE Guidance on respiratory protective equipment at work HSG53 https://www.hse.gov.uk/pubns/books/hsg53.htm

HSE Guidance on respiratory protective equipment (RPE) fit testing INDG479 https://www.hse.gov.uk/pubns/indg479.pdf

HSE website page on fit testing https://www.hse.gov.uk/respiratory-protective-equipment/fit-testing-basics.htm

HSE guidance on fit testing during COVID-19 https://www.hse.gov.uk/coronavirus/ppe-face-masks/face-mask-ppe-rpe.htm

British Industry Safety Federation Fit2fit companion guides https://www.bsif.co.uk/fit2fit-companions-released/

For peer review only - http://bmjopen.bmj.com/site/about/guidelines.xhtml

Revised Standards for Quality Improvement Reporting Excellence (SQUIRE 2.0)
September 15, 2015

Text Section and Item Name	Section or Item Description
**Notes to authors**	• The SQUIRE guidelines provide a framework for reporting new knowledge about how to improve healthcare<br>• The SQUIRE guidelines are intended for reports that describe system level work to improve the quality, safety, and value of healthcare, and used methods to establish that observed outcomes were due to the intervention(s).<br>• A range of approaches exists for improving healthcare. SQUIRE may be adapted for reporting any of these.<br>• Authors should consider every SQUIRE item, but it may be inappropriate or unnecessary to include every SQUIRE element in a particular manuscript.<br>• The SQUIRE Glossary contains definitions of many of the key words in SQUIRE.<br>• The Explanation and Elaboration document provides specific examples of well-written SQUIRE items, and an in-depth explanation of each item.<br>• Please cite SQUIRE when it is used to write a manuscript.
**Title and Abstract**	
**1. Title**	**Page 1, Line 5**<br>Factors affecting successful fit testing of Respiratory Protective Equipment during COVID-19: A National Quality Improvement Initiative.

For peer review only

**2. Abstract**	**Page 2, Line 31**<br><br>**Introduction** Respiratory protective equipment has been a critical means to protect healthcare workers from COVID-19 infection. These devices are typically single use Filtering Facepiece Respirators, which are affixed to the face to prevent airborne transmission of the virus. There have been several reports of fitting issues within particular sub-groups of healthcare workers, although the factors affecting successful fitting of masks are still largely unknown.<br><br>**Objective** To evaluate factors affecting respirator fitting outcomes within a national health service during the COVID-19 pandemic.<br><br>**Methods** We conducted a secondary analysis of a national database involving fit testing outcomes collated between July to August 2020. Key demographics including age, gender, ethnicity and face measurements were compared to fitting outcome in a variety of respirator mask designs. A mixed-effects logistic regression model was used to determine the Odds Ratio for factors which affected fit testing outcome.<br><br>**Results** A total of 9,592 observations from 5,544 healthcare workers working with 47 UK Hospital Centres were included in the analysis. The mixed-effects logistic regression model identified that males experienced a significantly ($p<0.05$) higher fit test success than females (OR = 1.57; 95% CI: 1.31 to 1.88). Those with non-white ethnicities demonstrated lower odds of successful mask fitting, with black (OR = 0.66; 95% CI: 0.51 to 0.86), Asian (OR = 0.60; 95% CI: 0.50 to 0.71) and mixed (OR = 0.79; 95% CI: 0.64 to 0.99) ethnic groups all being statistically significant ($p<0.05$). There were significant differences ($p<0.05$) between mask models, when controlling for sex, age and ethnicity.<br><br>**Conclusion** During the early phase of COVID-19, females and non-White ethnicities were less likely to have a successful respirator mask fitting in England, with a high degree in variability regarding mask model fitting outcomes. Due to the diverse workforce, healthcare settings require a range of masks to ensure successful fitting outcomes. Further improvements with industry representatives [illegible]
**Introduction**	*Why did you start?*
**3. Problem Description**	**Page 4, Line 94**<br><br>The use of respiratory protective equipment (RPE) is vital in the prevention of nosocomial viral transmission for conditions such as influenza, SARS and SARS-CoV-2 (COVID-19).

For peer review only

**4. Available knowledge**	**Page 4, line 104**<br><br>There are many factors which may influence successful RPE fitting [11]. These include the relationship between facial dimensions and RPE shape and the materials used within the mask which include both compliant and non-compliant designs [12]. Indeed, facial anthropometrics vary significantly across HCWs, between genders, age, ethnicities, occupational groups and on an individual basis. These differences will influence the performance of RPE and there is already some evidence, albeit contradictory, that overall protection varies with gender-based differences in facial dimension [13-16]. These findings are of clear relevance for the design and manufacture of respirators, which need to match the characteristics of HCWs who are predominantly female and often from ethnic minority communities [17]

For peer review only

5. **Rationale**	**Page 4, line 118**<br>In the UK, the National Health Service (NHS) has purchased a variety of FFP3 RPE devices to protect frontline HCWs in response to SARS-CoV-2 (COVID-19) as part of their pandemic preparedness stockpile. However, it is not known how key demographics of healthcare workers effects fitting outcomes of FFP3 masks.
6. **Specific aims**	**Page 4, line 124**<br>Aim is to analyze data from a quality improvement initiative to answer the following research questions:<br>1. Do intrinsic factors, namely, gender identity, ethnic background, age and BMI affect the success rates of FFP3 mask fitting?<br>2. Is there a variation in performance of specific mask models in terms of fit test success over the number of attempts?<br>3. Do specific mask models match subject classification in terms of gender and ethnic group?<br>4. Do individual facial measurement parameters reflect the likelihood of [illegible]
**Methods**	*What did you do?*
7. **Context**	**Page 5, Line 133**<br>This retrospective evaluation study, involving National Health Service (NHS) hospitals in England, used data detailing fit test outcomes in all healthcare workers in the early stages of the COVID-19 pandemic.
8. **Intervention(s)**	**Page 5, line 135**<br>Fit testing of Filtering Facepiece Respirators (FFP3 masks) on a cohort of healthcare workers in England, working in the national health service.
**9. Study of the Intervention(s)**	**Page 5, line 144**<br>The outcomes is the successful fitting of the FFP3 mask from fit testing clinics in the NHS.

For peer review only

**10. Measures**	**Page 5, line 149**<br>Data included the type and make of RPE models used and success or failure of fitting according to two standardised and prescribed methods from the Health and Safety Executive, namely the qualitative fit test (QLFT) or the quantitative fit test (QNFT) [18,19]. This was conducted with a ToolKit provided (e-Appendix) to each hospital trust, through which details of demographics were collected, including gender, ethnic background, age and BMI. In the latter case, BMIs were categories using the World Health Organization (WHO) classifications as underweight (<18.5 kg/m^2), optimal (18.5-25 kg/m^2), overweight (25-30 kg/m^2) and obese (>30 kg/m^2) [20]. In addition, five facial anthropometrical measurements were estimated with a paper-based ruler, namely, facial length, nasal length and protrusion, alar and bi-ocular width.
**11. Analysis**	**Page 6, line 186**<br>Cleaned data was imported into Stata (Stata 16.0, StataCorp LLC, USA), where a basic model assessment was performed using the Bayesian information criterion (BIC). The model with the lowest BIC value was selected incorporating the mask model, gender, ethnicity and age. Following this evaluation, a mixed-effects logistic regression model was used to determine the ORs and their 95% confidence intervals (95% CI) for factors which affected fit testing outcome using a collated data set across each of the five fit test attempts. Analysis was conducted while adjusting for multiple risk factors and potential confounders. This approach accounted for nested data (as several, different masks were fitted to the same person) and for within-subject correlation by means of a random effect for subject.
**12. Ethical Considerations**	**Page 17, line 442**<br>Institutional ethics was approved to retrospectively analyse an anonymised version of the data set (ERGO-65166). Data was confidentially shared with the research group at the University of Southampton (UoS) and stored in accordance with the Data Protection Act 2018.
**Results**	*What did you find?*
**13. Results**	**Page 7, line 201**<br>A total of 9,592 observations from 5,512 healthcare workers were included in the regression model. Most of the individuals, approximately 78% of the total for all attempts, were aged between 24 and 54 years of age. Those that were younger (18-24 years) represented 9% of the cohort, whereas those in the older categories (up to 74 years of age) represented 13% of the participants.<br>The mixed-effects logistic regression model identified that males experienced a significantly ($p<0.05$) higher fit test success than females (OR = 1.57; 95% CI: 1.31 to 1.88). Those with non-white ethnicities demonstrated lower odds of successful mask fitting, with black (OR = 0.66; 95% CI: 0.51 to 0.86), Asian (OR = 0.60; 95% CI: 0.50 to 0.71) and mixed (OR = 0.79; 95% CI: 0.64 to 0.99) ethnic groups all being statistically significant ($p<0.05$). There were significant differences ($p<0.05$) between mask models, when controlling for sex, age and ethnicity.

For peer review only

Discussion	*What does it mean?*
**14. Summary**	**Page 14, line 309**<br>During the early phase of COVID-19, females and non-White ethnicities were less likely to have a successful respirator mask fitting in England, with a high degree in variability regarding mask model fitting outcomes.

For peer review only

**15. Interpretation**	**Page 14, line 336**<br>Due to the diverse workforce, healthcare settings require a range of masks to ensure successful fitting outcomes. Further improvements with industry representatives and standards committees is required to ensure mask designs are fit for purpose.
**16. Limitations**	**Page 15, line 350**<br>This secondary analysis of quality improvement data was limited by the nature in which individual hospital trust collected and documented fit testing. Indeed, a small proportion of data was omitted during the data cleaning process in order to conduct robust analysis. The results of this retrospective analysis are only reflective of the time period of investigation (July-August 2020), with improvements to mask designs and fitting processes evident during the latter stages of the pandemic. The data presented in the present study is also a product of the international demands on PPE supply chain 3, with a diverse range of masks included in the data set. As a result, RPE in different English hospitals was variable and procurement dependent. In addition, implementing a comprehensive fit-testing program is a financial and logistical challenge 43, limiting the feasibility to test all HCWs on different FFP3 mask models.
**17. Conclusions**	**Page 16, line 389**<br>This secondary analysis of fit testing outcomes derived from a multi-centre data set revealed a range of FFP3 mask models were procured in the early stages (July and August 2020) of the pandemic to protect HCWs involved in managing COVID-19 patients. The data identified distinct trends in fit testing outcomes related to both gender and ethnicity. Indeed, white males were the most likely to yield a successful fit test outcome and ethnic minority females the least likely. In many cases, repeat fit testing with different respirator models was required to ensure individual staff had a correctly fitting device. Further research is needed to provide improvements in the fit testing approach resulting in a more intelligent selection process. In addition, collaborative working with industry and standards representatives is required to optimise RPE designs to accommodate a wide range of face shapes incorporating different genders and ethnicities. In addition, mask designs and material interfaces need to support the protection of skin health for those who require devices for prolonged periods.
**Other information**	
**18. Funding**	**Page 16, line 406**<br>The secondary analysis of data was supported by UK Research and Innovation (UKRI) as part of a funded project "A Bio-Engineering approach for the SAFE design and fitting of Respiratory Protective Equipment (BE-SAFE RPE)" (Ref EP/V045563/1).

For peer review only

Table 2. Glossary of key terms used in SQUIRE 2.0. This Glossary provides the intended meaning of selected words and phrases as they are used in the SQUIRE 2.0 Guidelines. They may, and often do, have different meanings in other disciplines, situations, and settings .

Assumptions
Reasons for choosing the activities and tools used to bring about changes in healthcare services at the system level.

Context
Physical and sociocultural makeup of the local environment (for example, external environmental factors, organizational dynamics, collaboration, resources, leadership, and the like), and the interpretation of these factors ("sense-making") by the healthcare delivery professionals, patients, and caregivers that can affect the effectiveness and generalizab ility of intervention(s).

Ethical aspects
The value of system-level initiatives relative to their potential for harm, burden, and cost to the stakeholders. Potential harms particularly associated with efforts to improve the quality, safety, and value of healthcare services include opportunity costs, invasion of privacy, and staff distress resulting from disclosure of poor performance.

Generalizability
The likelihood that the interve ntio n(s) in a particular report would produce similar results in other settings, situations, or environments (also referred to as external validity).

Healthcare improvement
Any systematic effort intended to raise the quality, safety, and value of healthcare services, usually done at the system level. We encourage the use of this phrase rather than "quality improvement," which often refers to more narrowly defined approaches.

Inferences
The meaning of findings or data, as interpreted by the stakeholders in healthcare services – improvers, healthcare delivery professionals, and/or patients and families

Initiative
A broad term that can refer to organization-wide programs, narrowly focused projects, or the details of specific interventions (for example, planning, execution, and assessment)

Internal validity
Demonstrable, credible evidence for efficacy (meaningful impact or change) resulting from introduction of a specific intervention into a particular healthcare system.

Intervention(s)
The specific activities and tools introduced into a healthcare system with the aim of changing its performance for the better. Complete description of an intervention includes its inputs, internal activities, and outputs (in the form of a logic model, for example), and the mechanism(s) by which these components are expected to produce changes in a ystem's performance.

Opportunity costs

For peer review only

Loss of the ability to perform other tasks or meet other responsibilities resulting from the diversion of resources needed to introduce, test, or sustain a particular improvement initiative

Problem
Meaningful disruption, failure, inadequacy, distress, confusion or other dysfunction in a healthcare service delivery system that adversely affects patients, staff, or the system as a whole, or that prevents care from reaching its full potential

Process
The routines and other activities through which healthcare services are delivered

Rationale
Explanation of why particular intervention(s) were chosen and why it was expected to work, be sustainable, and be replicable elsewhere.

Systems
The interrelated structures, people, processes, and activities that together create healthcare services for and with individual patients and populations. For example, systems exist from the personal self-care system of a patient, to the individual provider-patient dyad system, to the microsystem, to the macrosystem, and all the way to the market/social/insurance system. These levels are nested within each other.

Theory or theories
Any "reason-giving" account that asserts causal relationships between variables (causal theory) or that makes sense of an otherwise obscure process or situation (explanatory theory). Theories come in many forms, and serve different purposes in the phases of improvement work. It is important to be explicit and well-founded about any informal and formal theory (or theories) that are used.

For peer review only

BMJ Open

Retrospective evaluation of factors affecting successful fit testing of Respiratory Protective Equipment during the early phase of COVID-19.

Journal:	*BMJ Open*
Manuscript ID	bmjopen-2022-065068.R1
Article Type:	Original research
Date Submitted by the Author:	21-Mar-2023
Complete List of Authors:	Caggiari, Silvia; University of Southampton, School of Health Sciences<br>Bader, Dan; University of Southampton, School of Health Sciences<br>Packman, Zoe; NHS England and NHS Improvement London<br>Robinson, Jane; NHS England and NHS Improvement London<br>Tranka , Sumeshni; NHS England and NHS Improvement London<br>Böhning, Dankmar ; University of Southampton, Southampton Statistical Sciences Research Institute<br>Worsley, Peter; University of Southampton
**Primary Subject Heading**:	Public health
Secondary Subject Heading:	Health policy, Health services research, Infectious diseases, Public health
Keywords:	COVID-19, OCCUPATIONAL & INDUSTRIAL MEDICINE, Clinical audit < HEALTH SERVICES ADMINISTRATION & MANAGEMENT

SCHOLARONE™ Manuscripts

I, the Submitting Author has the right to grant and does grant on behalf of all authors of the Work (as defined in the below author licence), an exclusive licence and/or a non-exclusive licence for contributions from authors who are: i) UK Crown employees; ii) where BMJ has agreed a CC-BY licence shall apply, and/or iii) in accordance with the terms applicable for US Federal Government officers or employees acting as part of their official duties; on a worldwide, perpetual, irrevocable, royalty-free basis to BMJ Publishing Group Ltd ("BMJ") its licensees and where the relevant Journal is co-owned by BMJ to the co-owners of the Journal, to publish the Work in this journal and any other BMJ products and to exploit all rights, as set out in our licence.

The Submitting Author accepts and understands that any supply made under these terms is made by BMJ to the Submitting Author unless you are acting as an employee on behalf of your employer or a postgraduate student of an affiliated institution which is paying any applicable article publishing charge ("APC") for Open Access articles. Where the Submitting Author wishes to make the Work available on an Open Access basis (and intends to pay the relevant APC), the terms of reuse of such Open Access shall be governed by a Creative Commons licence – details of these licences and which Creative Commons licence will apply to this Work are set out in our licence referred to above.

Other than as permitted in any relevant BMJ Author's Self Archiving Policies, I confirm this Work has not been accepted for publication elsewhere, is not being considered for publication elsewhere and does not duplicate material already published. I confirm all authors consent to publication of this Work and authorise the granting of this licence.

For peer review only

BMJ Open

Title: Retrospective evaluation of factors affecting successful fit testing of Respiratory Protective Equipment during the early phase of COVID-19

Authors: Silvia Caggiari [1], Dan L. Bader [1], Zoe Packman [2], Jane Robinson [2], Sue Tranka [2], Dankmar A. Böhning [3], Peter R. Worsley [1].

1. Clinical Academic Facility, School of Health Sciences, University of Southampton, Southampton, UK. SO17 1BJ
2. NHS England & Improvement, 3rd floor Skipton House, 80 London road, London, UK. SE1 6LH
3. Southampton Statistical Sciences Research Institute, University of Southampton, Southampton, UK, SO17 1BJ

Corresponding Author: Peter R Worsley. Clinical Academic Facility, School of Health Sciences, University of Southampton, Southampton, UK. SO17 1BJ

Email: P.R.Worsley@soton.ac.uk Tel: +44(0) 2381208287

Key Words: Personal Protective Equipment; COVID-19; Fitting; Occupational Health; Health Policy

Word Count: 3228

For peer review only

Abstract

Objectives: Respiratory protective equipment is critical to protect healthcare workers from COVID-19 infection, which includes Filtering Facepiece Respirators. There are reports of fitting issues within healthcare workers, although the factors affecting fitting outcomes are largely unknown. This study aimed to evaluate factors affecting respirator fitting outcomes.

Design: This is a retrospective evaluation study. We conducted a secondary analysis of a national database of fit testing outcomes in England between July to August 2020.

Settings: The study involves National Health Service (NHS) hospitals in England

Participants: A total of 9,592 observations regarding fit test outcomes from 5,604 healthcare workers were included in the analysis.

Intervention: Fit testing of Filtering Facepiece Respirators (FFP3) on a cohort of healthcare workers in England, working in the national health service.

Primary and secondary outcome measures: Primary outcome measure was the fit testing result i.e., pass or fail with a specific respirator. Key demographics including age, gender, ethnicity and face measurements of 5,604 healthcare workers were used to compare fitting outcomes.

Results: A total of 9,592 observations from 5,604 healthcare workers were included in the analysis. A mixed-effects logistic regression model was used to determine the factors which affected fit testing outcome. Results showed that males experienced a significantly ($p<0.05$) higher fit test success than females (OR = 1.51; 95% CI: 1.27 to 1.81). Those with non-white ethnicities demonstrated significantly lower odds of successful respirator fitting; Black (OR = 0.65; 95% CI: 0.51 to 0.83), Asian (OR = 0.62; 95% CI: 0.52 to 0.74) and Mixed (OR = 0.60; 95% CI: 0.45 to 0.79

Conclusion: During the early phase of COVID-19, females and non-White ethnicities were less likely to have a successful respirator fitting. Further research is needed to design new respirators which provide equal opportunity for comfortable, effective fitting of these devices.

For peer review only - http://bmjopen.bmj.com/site/about/guidelines.xhtml

Strength and limitations of the study

- Results from a national audit of fit testing outcomes from healthcare workers using Filtering Facepiece Respirators (FFP3)
- Data revealed significant differences between genders and ethnicities in fit testing outcomes.
- The study was limited to real world data with no controls, during the early stages of the pandemic
- No direct comparisons between FFP3 respirators could be made
- Our secondary analysis reveals significant bias in fitting outcomes and the need for improvements in FFP3 respirator designs and standards.

For peer review only

1. Introduction

The use of respiratory protective equipment (RPE) was vital in the prevention of airborne viral transmission for conditions such as influenza, SARS and SARS-CoV-2 (COVID-19). Indeed, a systematic review and meta-analysis demonstrated the use of N95 or FFP3respirators can reduce the risk of respiratory virus infection by 70%, suggesting respirator use offers significant protection against the transmission of such respiratory viruses [1]. In the specific context of COVID-19, the risk of infection was significantly reduced amongst healthcare workers (HCWs) wearing Filtering Facepiece Respirators, highlighting the importance of RPE in the current pandemic [2]. As a result, there was an international effort to procure Filtering Facepiece Respirators, often termed N95 respirators and FFP3 devices, creating an extreme demand on the associated supply chain [3 4].

Fit testing is used to minimise the inward leakage of the respirator when attached to the face and both qualitative fit tests (QLFT) and quantitative fit tests (QNFT) have been recommended [5 6]. However, respirators with sub-optimal fit are commonly used with a tightened strap used to create an airtight seal, thus endangering the health of the underlying skin on locations of the face [7]. Despite guidelines [8] and efforts to reduce skin damage, the prevalence of reported issues has remained high [9]. There are many factors which may influence successful RPE fitting [10]. These include the relationship between facial dimensions and RPE shape and the materials used within the respirator which include both compliant and non-compliant designs [11]. Facial anthropometrics differences may influence the performance of RPE and there is already some evidence, albeit contradictory, that overall protection varies with gender-based differences in facial dimension [12-15].

In England, the National Health Service (NHS) purchased a variety of FFP3 RPE devices to protect frontline HCWs in response to SARS-CoV-2 (COVID-19) as part of their pandemic preparedness stockpile. Recent studies in England have identified that male and White ethnicity healthcare workers are more likely to achieve RPE fit test success [16 17]. However, these were limited to a single healthcare centre limiting its generalisability. Thus, the present study aims to analyse this data retrospectively and evaluate the fitting outcomes of a national population of healthcare workers using several variations of FFP3 respirators. Objectives of the study were to assess whether:

1. Intrinsic factors, namely, gender identity, ethnic background, age and BMI affected the success rates of FFP3 respirator fitting.
2. Specific respirator testing methods (qualitative or quantitative) affected the success rates.
3. Individual facial measurement parameters reflected the likelihood of successful respirator fitting.

For peer review only

2. Methodology

This quality improvement study, involving National Health Service (NHS) hospitals in England, collated data regarding fit test outcomes in all healthcare workers. Each healthcare provider and individual participant were consented to take part in the study. Institutional ethics was approved by the Faculty of Environmental and Life Sciences (FELS) ethics committee to retrospectively analyse an anonymised version of the data set (ERGO-65166). Data was confidentially shared with the research group at the University of Southampton (UoS) and stored in accordance with the Data Protection Act 2018.

2.1 Fit testing data

Data included the success or failure of fitting or a range of FFP3 respirators (model A-G) according to two standardised and prescribed methods from the Health and Safety Executive, namely the qualitative fit test (QLFT) or the quantitative fit test (QNFT) [18 19]. This was conducted with a ToolKit provided (e-Appendix A) to each hospital trust, through which details of demographics were collected, including gender, ethnic background, age and BMI. In the latter case, BMIs were categories using the World Health Organization (WHO) classifications as underweight (<18.5 kg/m^2), optimal (18.5-25 kg/m^2), overweight (25-30 kg/m^2) and obese (>30 kg/m^2) [20]. In addition, five facial anthropometrical measurements were estimated with a paper-based ruler, namely, facial length, nasal length and protrusion, alar and bi-ocular width. Inclusion criteria consisted of hospital staff working in areas deemed necessary for FFP3 respirators . Healthcare workers were initially fitted with a respirator and assessed using the standardised tests. If they passed the respirator was retained, and those that failed had further attempts with other respirator models until successful (Figure 1). In some cases, HCWs were tested on multiple respirator variants even when successful fitting was initially achieved, this was conducted to counter any supply chain issues with specific respirator types.

[Insert Figure 1 near here]

Data collection was carried out in a 5 week period between July and August 2020. ToolKits were electronically distributed to a number of hospitals in England, identified with an anonymised code and responsible to name an experienced fit tester for the recruitment of staff members. All data were electronically collected, anonymised and stored securely within NHS England and Improvement.

2.2 Data analysis

Data detailed the result of up to five fit test attempts for FFP3 respirator respirators in a cohort of >5000 hospital staff. These data were reviewed to ensure consistency in the analytical methods. This

For peer review only - http://bmjopen.bmj.com/site/about/guidelines.xhtml

involved a cleansing process to minimise redundancy and remove outliers (from the paper-ruler facial measurements), missing or undeclared data. Subsequent review of the data included:

- Ethnic backgrounds initially categorized into 24 groups and subsequently into four major ethnic backgrounds, namely, White, Asian, Black and Mixed. Further analysis was conducted with respect to sub-groups within each ethnicities associated with the specific country of origin.
- A few individuals identified their gender as "Non binary", representing only 0.1% of the total cohort. These were excluded from the analysis, as they precluded an analysis of the effect of gender on success rates of respirator fitting.

2.3 Statistical Analysis

Cleaned data was imported into Stata (Stata 16.0, StataCorp LLC, USA), where a basic model assessment was performed using the Bayesian information criterion (BIC). The model with the lowest BIC value was selected incorporating the fit test type, respirator model, gender, ethnicity, and age. Following this evaluation, a mixed-effects logistic regression model was used to determine the ORs and their 95% confidence intervals (95% CI) for factors which affected fit testing outcome using a collated data set across each of the five fit test attempts. Analysis was conducted while adjusting for multiple risk factors and potential confounders. This approach accounted for nested data (as several, different respirators were fitted to the same person) and for within-subject correlation by means of a random effect for subject.

Explorative data analysis was also conducted to evaluate trends between fitting outcomes within different ethnicities and gender and BMI categories. In addition, a cumulative frequency approach was used to investigate the distribution of the facial measurements. Cluster analyses were conducted to identify any trends in the measurements associated with fit test outcome.

2.4 Patient and Public Involvement

Patients and public (healthcare workers) were consulted on the challenges associated with getting correctly fitting respirators and this formed the motivation for the quality improvement study. These were consulted regarding data collection forms and analysis**.**

3. Results

3.1 Overview of the data

A total of 9,592 observations from 5,604 healthcare workers were included in the regression model (132 data entries were removed from the quality control process). This included data from 47 NHS

For peer review only - http://bmjopen.bmj.com/site/about/guidelines.xhtml

Hospitals in England, where healthcare workers were fit tested with various respirator designs up to five times.

The number of fit attempts differed between individuals, with a steady decline from attempt 1 to 5. To review briefly, there were 5512, 2191, 1072, 560 and 257, in the first, second, third, fourth and fifth accumulative attempts, respectively (Table 1). There was a small increase in the proportion of Asian individuals between the first (20.3%) and fifth (27.2%) attempts, while the gender and BMI categories remained largely unchanged. The majority of the individuals (~80% of the total for all attempts) were identified to be in the middle two BMI classifications. Individuals with a BMI under 18 kg/m^2 represented the smallest proportion of the cohort (3-5%) in all five attempts. Most of the individuals, approximately 78% of the total for all attempts were aged between 24 and 54 years of age. Those that were younger (18-24 years) represented approximately 8% of the cohort, whereas those in the older categories (55+ years of age) represented approximately 12% of the participants.

Table 1: Summary of anthropometric data of participants of participants at each attempt of the fit test.

	**Total**	**Attempt 1**	**Attempt 2**	**Attempt 3**	**Attempt 4**	**Attempt 5**
Number of fit tests	9592	5512	2191	1072	560	257
Successful fitting	67.2%	72.2%	62.9%	58.4%	53.8%	63.8%
Gender						
Female	79.1%	79.6%	78.9%	78.6%	77.0%	74.7%
Male	20.9%	20.4%	21.1%	21.4%	23.0%	25.3%
Ethnicity						
White (Caucasian)	62.9%	64.3%	61.5%	62.0%	57.0%	61.9%
Asian/Asian British	22.3%	20.3%	23.1 %	24.8%	30.7%	27.2%
Black/African/Caribbean	8.4%	8.9%	8.8%	6.8%	6.6%	5.8%
Mixed/Multiple backgrounds	6.4%	6.5%	6.6%	6.3%	5.7%	5.1%
BMI						
Under 18.5 kg/m^2	3.6%	3.3%	3.6%	4.3%	4.6%	5.1%
18.5-25 kg/m^2	51.3%	50.2%	51.0%	53.3%	57.0%	58.0%
25-30 kg/m^2	30.6%	31.7%	30.8%	28.0%	26.8%	24.1%
Over 30 kg/m^2	14.5%	14.9%	14.6%	14.4%	11.6%	12.8%
Age						
18-24 years old	8.1%	8.7%	7.9%	7.2%	5.4%	6.2%
25-34 years old	32.3%	30.4%	32.4%	36.7%	38.9%	41.2%

For peer review only - http://bmjopen.bmj.com/site/about/guidelines.xhtml

35-44 years old	24.5%	24.3%	25.2%	24.3%	24.1%	22.6%
45-54 years old	23.2%	23.3%	23.1%	22.4%	23.9%	23.0%
55+ years old	11.9%	13.2%	11.3%	9.4%	7.7%	7.0%

3.2 Mixed-effects logistic regression model

The final model after adjusting for covariates in our multilevel mixed-effects logistic regression analysis indicated that the odds of experiencing a successful FFP3 respirator fitting were significantly ($p<0.05$) higher among those who were male (OR = 1.51; 95% CI: 1.27 to 1.81) compared to females. In addition, those with non-white ethnicities demonstrated lower odds of successful respirator fitting, with black (OR = 0.65; 95% CI: 0.51 to 0.83), Asian (OR = 0.62; 95% CI: 0.52 to 0.74) and mixed (OR = 0.60; 95% CI: 0.45 to 0.79) ethnic groups all being statistically significantly ($p<0.05$) when compared with the white ethnic group. Individuals who were assessed with a quantitative fit test had a significantly lower odds of successful fitting than those who performed a qualitative fit test (OR = 0.71; 95% CI: 0.61 to 0.82). HCW with a low BMI (below 18.5 kg/m^2) had a significant lower odds of success fitting compared to all the other categories (Table 2).

Table 2: Odds ratios (OR) of a successful FFP3 respirator fit test outcome and the corresponding 95% confidence intervals for each factor adjusted for all other factors using multilevel mixed-effects logistic regression.

**Factor**		**n**	**Odds Ratio**	**z**	**p> |z|**	**95% Confidence Interval**
**Gender**	Female	7583			*1 (base)*	
	Male	2009	1.514	4.61	**0.000**	1.269 to 1.806
**Ethnicity**	White	6034			*1 (base)*	
	Black	810	0.622	-5.36	**0.000**	0.523 to 0.741
	Asian	2133	0.653	-3.40	**0.001**	0.511 to 0.835
	Mixed	615	0.603	-3.54	**0.000**	0.456 to 0.798
**Type**	Quantitative	6801			*1 (base)*	
	Qualitative	2725	0.7	-4.71	**0.000**	0.619 to 0.821
**BMI**	18.5-25	4922			*1 (base)*	
	25-30	2935	1.161	1.91	**0.056**	0.996 to 1.353
	Over 30	1391	1.108	1.01	**0.312**	0.907 to 1.353

For peer review only

For peer review only - http://bmjopen.bmj.com/site/about/guidelines.xhtml

	Under 18.5	344	0.516	-3.66	**0.000**	0.362 to 0.735

LR test vs. logistic model: chibar2(01) = -5680.43   Prob >= chibar2 = 0.0000

The likelihood ratio test (last line Table 2) showed that the random effect for subject is strongly required, and the simple logistic regression approach would have been not sufficient here.

3.3 Explorative Data Analysis

Analysis of the 9,592 observations showed that 71% of the fit tests were qualitative, as opposed to ~28% of quantitative fit tests (Table 3). Closer examination of the data revealed that the test type did not influenc the relative proportion of success rates, with quantitative fit test showing similar success and failure rates. In addition, the type of fit test performed was equally distributed across categories of gender and ethnicity.

Table 3: Summary of. type of fit test across success rate, gender and ethnicity.

	Type of fit test	
	Qualitative	*Quantitative*
Total	71.4%	28.6%
Success rate		
Passed	69.4%	61.8%
Failed	30.6%	38.2%
Gender		
Female	78.0%%	82.1%
Male	22.0%	17.9%
Ethnicity		
White (Caucasian)	63.2%	61.2%
Asian/Asian British	8.1%	9.2%
Black/African/Caribbean	22.2%	22.3%
Mixed/Multiple backgrounds	6.5%	5.9%

Fit test outcomes also revealed that 60% of the HCWs were fit tested only once, as opposed to 4% who performed all the five attempts. In addition, data revealed that 17% of the individuals failed all the attempts performed, 60% passed one attempt, <15% of HCWs passed between 2 to 5 attempts.

For peer review only

With respect to ethnicity and gender, the group of White male participants yielded the highest success rates, with absolute differences of ≥ 4% compared to all other groups (Table 4). In a similar manner, the White females yielded a higher success rate than their corresponding ethnic comparators, with a difference of ≥ 5 %. Asian and Mixed background ethnic groups yielded the lowest success rate of 60.3% and 60.4%, respectively. Closer examination of the fit test outcome also revealed the lowest success rate of approximately 56% associated with Bangladeshi HCWs, although they only represent a small proportion (54/2133, ~2.5%) of the corresponding Asian cohort.

For peer review only

Table 4: The absolute differences in global fit test success rate with reference to gender and ethnicity. Created by the authors

Gender and Ethnic Group (participants, % success rate)	White Male	White Female	Asian/Asian British Male	Asian/Asian British Female	Black/African/ Caribbean Male	Black/African/ Caribbean Female	Mixed/Multiple backgrounds Male	Mixed/Multiple backgrounds Female
White Male (1046, 74.3%)	-	-5.8	-4.4	-14.0	-3.5	-10.8	-7.6	-13.9
White Female (4988, 68.5%)		-	1.4	-8.2	2.3	-5.0	-1.8	-8.1
Asian/Asian British Male (627, 69.9%)				-9.6	0.9	-6.4	-3.2	-9.5
Asian/Asian British Female (1506, 60.3%)				-	10.5	3.2	6.4	0.1
Black/African/Caribbean Male (168, 70.8%)					-	-7.3	-4.1	-10.4
Black/African/Caribbean Female (642, 63.5%)						-	3.2	-3.1
Mixed/Multiple backgrounds Male (168, 66.7%)							-	-6.3
Mixed/Multiple backgrounds Female (447, 60.4%)								-

For peer review only

3.5 Fit test outcomes according to facial measurements

Facial measurements were examined with respect to the total cohort of 5544 HCWs. The cumulative frequency trends for each of the facial parameters were found to be normally distributed and, as a result, parametric descriptors were used for analysis. Close examination of the data suggests that there was a limited discrimination between facial measurements and fit test outcomes, with a wide range of values attributed to individuals who both passed and failed at each attempt (e-Appendix B).

For peer review only

For peer review only - http://bmjopen.bmj.com/site/about/guidelines.xhtml

4. Discussion

A multi-centre quality improvement study was implemented to explore the factors associated with fitting outcomes for Filtering Facepiece Respirators (FFP3) during the early phase of COVID-19 (July and August 2020). Conducted through NHS England and Improvement, data describing the fitting outcomes from multiple respirator models were completed for over 5000 HCWs. The multivariate analysis revealed that women and non-white ethnicities yielded a significantly lower odds ratio of fit test success rates compared to males (Table 2). Gender-based differences have been associated with fit testing outcomes in a number of studies, the majority of which demonstrate that female participants yield a significantly lower RPE success rate, and as a result need a range of respirator models for successful fitting [12-16 21-28]. This is in contrast to a recent study in the England, where no gender differences were identified [15].

The present study also observed clear ethnicity-based differences in fit test outcomes, both for males and females, with Asian, Black and Mixed ethic background individuals having a lower fit test success rate when compared to White ethnicities. White males yielded the highest success rate (74%) as opposed to 60% of Asian and Mixed ethnicities females (Table 2 and 4). Small comparative studies have demonstrated lower pass rates for Black and Asian females [14 16 29 30]. In addition, studies of Asian populations have consistently yielded higher rates of fit test failure within Chinese, Koreans, Taiwanese and Iranian cohorts, further emphasising the importance of considering facial dimensions of the relevant population in RPE design [13 21 22 24-26 31-35]. These differences are likely to have resulted from the known effects of ethnicity on anthropometric facial features [36]. Therefore, the RPE models currently available may not provide comparable protection in a multi-ethnic workforce, thus disadvantaging those from minority groups. This implies that RPE designs evaluated during this study did not accommodate the heterogeneity in facial features across diverse user populations due to the limited nature of the in panels used for international standards e.g., EN-149.

It is also of note that fit testing success varied across the different BMI ranges, with the underweight category (<18.5kg/m^2) yielding the highest failure rates. Closer analysis of the present findings also revealed that 40% of individuals with low BMI were Asian, 52% of whom did not achieve success at any attempt at fit testing. This suggests that both ethnicity related anthropometrics of face shape and body mass index are contributing factors in fit test outcomes. Indeed, the current respirator fit panels do not accommodate those in the low BMI range, further evidence has shown that fitting will alter dependent on changes in an individual's weight [37]. Indeed, the soft tissue composition of the face could effect a change in the contact between the respirator and the underlying skin, with more bony prominences creating a less conforming surface from which respirators can create a seal.

For peer review only - http://bmjopen.bmj.com/site/about/guidelines.xhtml

The present study has identified significant differences between the quantitative and qualitative fit testing success rates (Table 2). This finding has also been demonstrated in other studies comparing the outcomes of these tests in cohorts evaluating the same respirator designs [10]. Here, there are a higher proportion of respirators passed using the qualitative methodology, and evidence that when a failure is observed in the quantitative test, a pass may still be achieved in the corresponding qualitative test for a given respirator [38]. Indeed, quantitative fit-testing has been defined as a gold standard and recommended to comply with international and national standards [10 39]. There are some limitations with qualitative fit testing. Firstly, it is a subjective test as it relies on the taste indicating the absence or presence of taste. Additionally, the test hood may not be tolerated by HCWs with claustrophobia. It is also possible that some HCWs with increased anxiety may intentionally or unintentionally fail the fit test (indicating a leak) during a pandemic [10].

This highlights the importance of an effective fitting process prior to use. Indeed, there is the need to support standard methods with intelligent fitting algorithms able to characterise the goodness of fit in an objective manner and predict respirator fitting, to provide individuals with safe and effective respirator [40].

4.1 Limitations

This secondary analysis of quality improvement data was limited by the nature in which individual hospital trust collected and documented fit testing. Indeed, a small proportion of data was omitted during the data cleaning process in order to conduct robust analysis. This was performed using defined criteria and enabled robust analysis, for example removing miss-labelled data or entries which did not include key parameters e.g., test type or ethnicity. The results of this retrospective analysis are only reflective of the time period of investigation (July-August 2020), with improvements to respirator designs and fitting processes evident during the latter stages of the pandemic. The data presented in the present study is also a product of the international demands on PPE supply chain [3], with a diverse range of respirator s included in the data set. As a result, RPE in different English hospitals was variable and procurement dependent. In addition, implementing a comprehensive fit-testing program is a financial and logistical challenge [41], limiting the feasibility to test all HCWs on different FFP3 respirator models. Comparisons between respirators could not be performed as there was no random allocation of respirator types across the fit testing attempts. The anthropometric data was limited by the use of paper-based rulers, with limited accuracy and reliability. Given the findings of gender and ethnicity differences, further evaluation of anthropometrics using accurate scanning technologies are required.

For peer review only

4.2 Clinical Implications

In practice, poorly fitted RPE designs impede both functional capacity and user safety [42 43]. Widespread concerns in areas of RPE fit-test access, availability and training have been highlighted during the pandemic [44 45]. Notably, for HCWs using RPE for prolonged periods, skin damage has been reported with a variable prevalence of between 42% and 97%. This has been attributed to ill-fitting RPE and may account for higher rates of adverse reactions within Ethnic minorities individuals [9 46-48]. In many countries there have been significant improvements in the supply chain of RPE devices and a range of models is available to many healthcare institutions. Further research into the design and fitting of RPE must consider the demographics of the healthcare workforce including the diverse range of ethnicities, ages and genders. Design panels incorporating established data sets of different sub-groups of demographics could inform new designs and international standards (e.g. ISO/DIS 16976-6) by which FFP3 respirator s can be manufactured. Healthcare institutions should maintain a range of RPE devices to accommodate successful fitting for their diverse workforce and data shared with industrial representatives to ensure the available designs are fit for purpose.

5. Conclusion

This secondary analysis of respirator fit testing outcomes revealed distinct trends in fit testing outcomes related to both gender and ethnicity. Indeed, white males were the most likely to yield a successful fit test outcome and ethnic minority females the least likely. In many cases, repeat fit testing with different respirator models was required to ensure individual staff had a correctly fitting device. Further research is needed to provide improvements in the fit testing approach resulting in a more intelligent selection process, and a more expansive selection of respirators is required to meet the diverse population of healthcare workers.

Acknowledgements

We would like to thank all the participating hospital trusts support data collection during the Quality Improvement Initiative.

Funding Statement

This study was commissioned by NHS England and Improvement. The secondary analysis of data was supported by UK Research and Innovation (UKRI) as part of a funded project "A Bio-Engineering approach for the SAFE design and fitting of Respiratory Protective Equipment (BE-SAFE RPE)" (Ref EP/V045563/1).

For peer review only - http://bmjopen.bmj.com/site/about/guidelines.xhtml

Competing interest

There are no conflicts of interest for any of the authors in this manuscript.

Data Sharing

Technical appendix, statistical code, and dataset available from the Pure repository, DOI:10.5258/SOTON/D2381.

Author Contribution

SC was responsible for conceptualisation, data acquisition, analysis, interpretation, writing original draft, review, and editing. PW was responsible for conceptualisation, funding acquisition, supervision, writing critical review and editing. ZP, JR, ST were responsible for data acquisition for the study, constant coordination, writing critical review and editing. DLB was responsible for conceptualisation, funding acquisition, data acquisition, supervision, writing critical review and editing. DAB was responsible for statistical analysis and writing.

Figure Captions

Figure 1: Flow chart depicting the respirator testing process and repeat assessments which contributed to the data set.

References

1. Liang M, Gao L, Cheng C, et al. Efficacy of face mask in preventing respiratory virus transmission: A systematic review and meta-analysis. *Travel Med Infect Dis* 2020;36:101751. doi: https://doi.org/10.1016/j.tmaid.2020.101751 [published Online First: 2020/05/31]

2. Li Y, Liang M, Gao L, et al. Face masks to prevent transmission of COVID-19: A systematic review and meta-analysis. *Am J Infect Control* 2020 doi: https://doi.org/10.1016/j.ajic.2020.12.007 [published Online First: 2020/12/22]

3. Feinmann J. PPE: what now for the global supply chain? *BMJ* 2020;369:m1910. doi: https://doi.org/10.1136/bmj.m1910

4. Miller FA, Young SB, Dobrow M, et al. Vulnerability of the medical product supply chain: the wake-up call of COVID-19. *BMJ Qual Saf* 2021;30(4):331-35. doi: https://doi.org/10.1136/bmjqs-2020-012133 [published Online First: 2020/11/04]

For peer review only - http://bmjopen.bmj.com/site/about/guidelines.xhtml

5. NIOSH. RCT-APR-STP-0068: Total Inward Leakage Test for Half-mask Air-purifying Particulate Respirators. *42 CFR Part 84, Subpart K: National Institute for Occupational Safety and Health* 2009

6. OSHA. 1910.134: Respiratory protection. App A: Fit testing procedures (mandatory). Subpart I: Personal Protective Equipment: Occupational Safety and Health Administration.

7. Worsley PR, Spratt F, Bader DL. COVID19: Challenging tissue viability in both patients and clinicians. *Journal of Tissue Viability* 2020;29(3):153-54. doi: https://doi.org/10.1016/j.jtv.2020.06.003

8. NHS England and NHS Improvement. Helping prevent facial skin damage beneath personal protective equipment. 2019

9. Abiakam N, Worsley P, Jayabal H, et al. Personal protective equipment related skin reactions in healthcare professionals during COVID-19. *Int Wound J* 2021;18(3):312-22. doi: 10.1111/iwj.13534 [published Online First: 2021/01/29]

10. Regli A, Sommerfield A, von Ungern-Sternberg BS. The role of fit testing N95/FFP2/FFP3 masks: a narrative review. *Anaesthesia* 2021;76(1):91-100. doi: https://doi.org/10.1111/anae.15261

11. Lawrence RB, Duling MG, Calvert CA, et al. Comparison of performance of three different types of respiratory protection devices. *J Occup Environ Hyg* 2006;3(9):465-74. doi: https://doi.org/10.1080/15459620600829211 [published Online First: 2006/07/22]

12. Oestenstad RK, Perkins LL. An assessment of critical anthropometric dimensions for predicting the fit of a half-mask respirator. *American Industrial Hygiene Association Journal* 1992;53(10):639-44. doi: https://doi.org/10.1080/15298669291360283

13. Han D-H, Choi K-L. Facial Dimensions and Predictors of Fit for Half-Mask Respirators in Koreans. *AIHA Journal* 2003;64(6):815-22. doi: https://doi.org/10.1080/15428110308984877

14. Manganyi J, Wilson KS, Rees D. Quantitative Respirator Fit, Face Sizes, and Determinants of Fit in South African Diagnostic Laboratory Respirator Users. *Ann Work Expo Health* 2017;61(9):1154-62. doi: https://doi.org/10.1093/annweh/wxx077

15. Green S, Gani A, Bailey M, et al. Fit-testing of respiratory protective equipment in the UK during the initial response to the COVID-19 pandemic. *Journal of Hospital Infection* 2021;113:180-86. doi: https://doi.org/10.1016/j.jhin.2021.04.024

16. Carvalho CYM, Schumacher J, Greig PR, et al. Prospective observational study of gender and ethnicity biases in respiratory protective equipment for healthcare workers in the COVID-19 pandemic. *BMJ Open* 2021;11(5):e047716. doi: 10.1136/bmjopen-2020-047716

17. Ascott A, Crowest P, de Sausmarez E, et al. Respiratory personal protective equipment for healthcare workers: impact of sex differences on respirator fit test results. *Br J Anaesth* 2021;126(1):e48-e49. doi: 10.1016/j.bja.2020.10.016 [published Online First: 2020/11/14]

For peer review only

18. Brienza D, Vallely J, Karg P, et al. An MRI investigation of the effects of user anatomy and wheelchair cushion type on tissue deformation. *Journal of Tissue Viability* 2018;27(1):42-53. doi: https://doi.org/10.1016/j.jtv.2017.04.001
19. Bogie KM, Nuseibeh I, Bader DL. Early progressive changes in tissue viability in the seated spinal cord injured subject. *Paraplegia* 1995;33:141-47.
20. Bakhsh TM. Results of unicompartmental knee replacement. *Saudi Medical Journal* 2007;28(7):1062-64.
21. Han DH. Fit factors for quarter masks and facial size categories. *The Annals of Occupational Hygiene* 2000;44(3):227-34. doi: https://doi.org/10.1016/S0003-4878(99)00087-3
22. Kim H, Han DH, Roh YM, et al. Facial anthropometric dimensions of Koreans and their associations with fit of quarter-mask respirators. *Ind Health* 2003;41(1):8-18. doi: https://doi.org/10.2486/indhealth.41.8 [published Online First: 2003/04/04]
23. McMahon E, Wada K, Dufresne A. Implementing fit testing for N95 filtering facepiece respirators: Practical information from a large cohort of hospital workers. *American Journal of Infection Control* 2008;36(4):298-300. doi: https://doi.org/10.1016/j.ajic.2007.10.014
24. Yu Y, Jiang L, Zhuang Z, et al. Fitting Characteristics of N95 Filtering-Facepiece Respirators Used Widely in China. *PLOS ONE* 2014;9(1):e85299. doi: https://doi.org/10.1371/journal.pone.0085299
25. Honarbakhsh M, Jahangiri M, Ghaem H, et al. Qualitative Fit Testing of Medium - Size N95/FFP2 Respirators on Iranian Health Care Workers. *Health Scope* 2018;7(4):e62884. doi: https://doi.org/10.5812/jhealthscope.62884 [published Online First: 2018-05-29]
26. Huh YJ, Jeong HM, Lim J, et al. Fit Characteristics of N95 Filtering Facepiece Respirators and the Accuracy of the User Seal Check among Koreans. *Infect Control Hosp Epidemiol* 2018;39(1):104-07. doi: https://doi.org/10.1017/ice.2017.271 [published Online First: 2018/01/19]
27. Oestenstad RK, Dillion HK, Perkins LL. Distribution of Faceseal Leak Sites on a Half-Mask Respirator and Their Association with Facial Dimensions. *American Industrial Hygiene Association Journal* 1990;51(5):285-90. doi: https://doi.org/10.1080/15298669091369664
28. Chopra J, Abiakam N, Kim H, et al. The influence of gender and ethnicity on facemasks and respiratory protective equipment fit: a systematic review and meta-analysis. *BMJ Glob Health* 2021;6(11) doi: http://doi.org/10.1136/bmjgh-2021-005537
29. Brazile WJ, Buchan RM, Sandfort DR, et al. Respirator Fit and Facial Dimensions of Two Minority Groups. *Applied Occupational and Environmental Hygiene* 1998;13(4):233-37. doi: https://doi.org/10.1080/1047322X.1998.10390073
30. Spies A, Wilson KS, Ferrie R. Respirator fit of a medium mask on a group of South Africans: a cross-sectional study. *Environ Health* 2011;10:17. doi: https://doi.org/10.1186/1476-069x-10-17 [published Online First: 2011/03/17]

For peer review only - http://bmjopen.bmj.com/site/about/guidelines.xhtml

31. Lin YC, Chen CP. Characterization of small-to-medium head-and-face dimensions for developing respirator fit test panels and evaluating fit of filtering facepiece respirators with different faceseal design. *PLoS One* 2017;12(11):e0188638. doi: https://doi.org/10.1371/journal.pone.0188638 [published Online First: 2017/11/28]

32. Foereland S, Robertsen O, Hegseth MN. Do Various Respirator Models Fit the Workers in the Norwegian Smelting Industry? *Saf Health Work* 2019;10(3):370-76. doi: https://doi.org/10.1016/j.shaw.2019.06.004 [published Online First: 2019/09/10]

33. Fakherpour A, Jahangiri M, Seif M. Qualitative fitting characteristics of filtering face-piece respirators on Iranian people. *J Environ Health Sci Eng* 2020;18(2):587-97. doi: https://doi.org/10.1007/s40201-020-00484-x

34. Zhang X, Jia N, Wang Z. The relationship between the filtering facepiece respirator fit and the facial anthropometric dimensions among Chinese people. *Ind Health* 2020;58(4):318-24. doi: https://doi.org/10.2486/indhealth.2019-0158

35. Fakherpour A, Jahangiri M, Seif M, et al. Quantitative fit testing of filtering face-piece respirators during the COVID-19 pandemic reveals anthropometric deficits in most respirators available in Iran. *Journal of Environmental Health Science and Engineering* 2021;19(1):805-17. doi: https://doi.org/10.1007/s40201-021-00648-3

36. Zhuang Z, Landsittel D, Benson S, et al. Facial anthropometric differences among gender, ethnicity, and age groups. *Ann Occup Hyg* 2010;54(4):391-402. doi: https://doi.org/10.1093/annhyg/meq007

37. Roberge R, Zhuang Z, Stein L. Association of body mass index with facial dimensions for defining respiratory fit panels. American Industrial Hygiene Conference and Exposition. Chicago, Illinois, 2006.

38. Hon CY, Danyluk Q, Bryce E, et al. Comparison of qualitative and quantitative fit-testing results for three commonly used respirators in the healthcare sector. *J Occup Environ Hyg* 2017;14(3):175-79. doi: 10.1080/15459624.2016.1237030 [published Online First: 2016/10/09]

39. Hon C-Y, Danyluk Q, Bryce E, et al. Comparison of qualitative and quantitative fit-testing results for three commonly used respirators in the healthcare sector. *Journal of Occupational and Environmental Hygiene* 2017;14(3):175-79. doi: 10.1080/15459624.2016.1237030

40. Caggiari S, Keenan B, Bader DL, et al. A combined imaging, deformation and registration methodology for predicting respirator fitting. *PLOS ONE* 2022;17(11):e0277570. doi: 10.1371/journal.pone.0277570

41. Wilkinson IJ, Pisaniello D, Ahmad J, et al. Evaluation of a large-scale quantitative respirator-fit testing program for healthcare workers: survey results. *Infect Control Hosp Epidemiol* 2010;31(9):918-25. doi: https://doi.org/110.1086/655460 [published Online First: 2010/07/28]

For peer review only - http://bmjopen.bmj.com/site/about/guidelines.xhtml

42. Sathasivam S, Walker PS. The conflicting requirements of laxity and conformity in total knee replacement. *J Biomech* 1999;32(3):239-47. doi: https://doi.org/10.1016/s0021-9290(98)00139-0 [published Online First: 1999/03/27]

43. Fidler H. PPE: 'one size fits all' design is a fallacy that's putting female health staff at risk. . *Nursing Standard* 2020

44. RCN. Second Personal Protective Equipment Survey of UK Nursing Staff Report: Use and availability of PPE during the COVID-19 pandemic. 2020

45. Gilroy R. PPE designed for rugby players not BME women, warns MP. *Nursing Times* 2020

46. Zhao Y, Liang W, Luo Y, et al. Personal protective equipment protecting healthcare workers in the Chinese epicentre of COVID-19. *Clin Microbiol Infect* 2020;26(12):1716-18. doi: https://doi.org/10.1016/j.cmi.2020.07.029 [published Online First: 2020/07/28]

47. Jiang Q, Song S, Zhou J, et al. The Prevalence, Characteristics, and Prevention Status of Skin Injury Caused by Personal Protective Equipment Among Medical Staff in Fighting COVID-19: A Multicenter, Cross-Sectional Study. *Adv Wound Care (New Rochelle)* 2020;9(7):357-64. doi: https://doi.org/10.1089/wound.2020.1212 [published Online First: 2020/04/23]

48. Lan J, Song Z, Miao X, et al. Skin damage among health care workers managing coronavirus disease-2019. *J Am Acad Dermatol* 2020;82(5):1215-16. doi: https://doi.org/10.1016/j.jaad.2020.03.014 [published Online First: 2020/03/17]

For peer review only - http://bmjopen.bmj.com/site/about/guidelines.xhtml

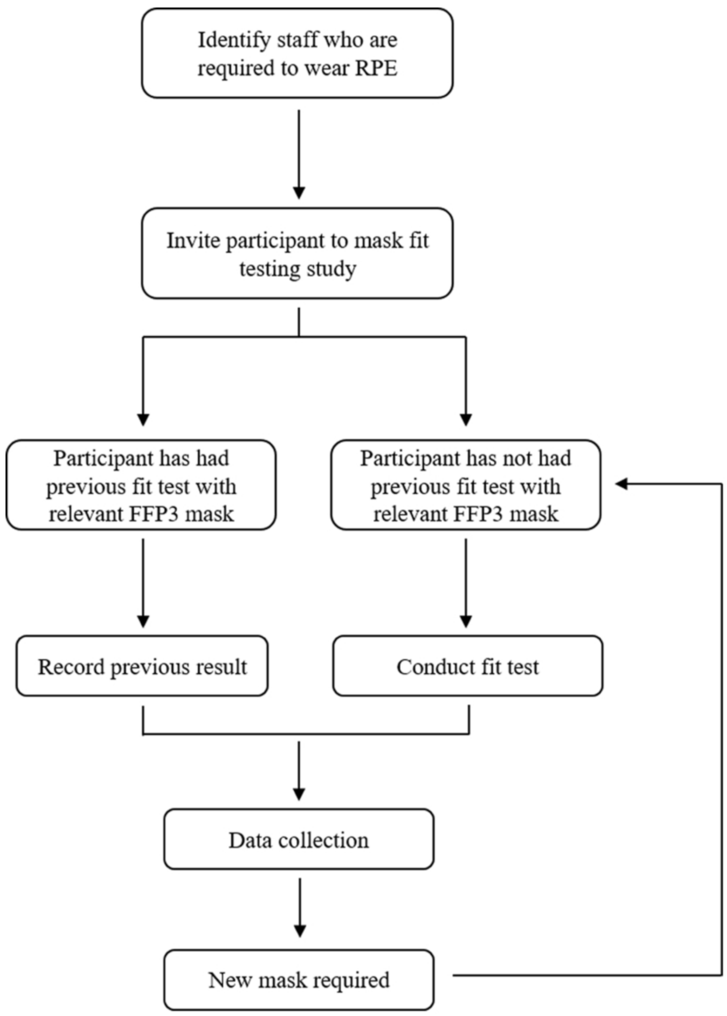

Figure 1: Schematic of mask fitting and data collection process.

1322x1869mm (96 x 96 DPI)

Appendix A

Learning from NHS England and NHS Improvement Quality Improvement Programme: FFP3 mask fit testing

A short toolkit for board members, infection prevention and control teams, managers and staff in all healthcare providers responsible for delivering an ongoing fit test training programme

Background to this toolkit

This short toolkit is designed to support NHS organisations to improve compliance with and uptake of FFP3 fit testing, improve staff experience, and enhance staff safety and well-being. It draws upon work undertaken by NHS England and NHS Improvement, and advice from other organisations, including the Health and Safety Executive, to identify best practice to support fit testing of FFP3 respirator masks and staff safety.

Summary of successful fit testing programmes

Using a rapid quality improvement approach, NHS England and NHS Improvement worked with 11 NHS organisations to identify the steps needed to ensure all staff required to wear an FFP3 mask are successfully fit tested with an appropriate mask. The improvement project enabled the participating organisations to rapidly identify barriers and implement solutions using small-scale tests of change. Through the course of the project several common themes emerged as being critical to the success of the fit testing process.

Collaboration

Departments key for facilitating successful fit testing include procurement, trust education and communication teams, health and safety teams, as well as staff networks, employee wellbeing, and occupational health teams. This ensures an integrated approach that coordinates supply, implementation, and education to deliver a programme with staff safety at its core.

Recommendation:

- Consider who is on the team, which other departments need to be involved and any additional support required for the delivery of the fit testing programme.

Education and communication with clinical and non-clinical staff

Evidence has suggested that some staff don’t understand the difference between a fit check and a fit test. Several trusts identified gaps in understanding and embarked on staff education programmes which covered guidance on preparing for a fit test. This involved the use of posters, dedicated intranet pages, and regular bulletins. One trust set up a dedicated email account for advice about fit testing, and empowered fit test leads to have a proactive role in engaging departmental leads in understanding the importance of fit testing for staff. “PPE marshals” supported and trained staff in correct donning and doffing

For peer review only

procedures and monitoring adherence within the work setting following fit testing i.e. that fit checks were carried out and PPE was appropriately worn.

Staff experience was evaluated through the use of wellbeing charts, focus groups, surveys and analysis of staff journeys through the fit test process.

Recommendation:

- Ensure appropriate education and communication is in place for staff about the reasons for fit testing, and the process.

Accessibility of fit testing, including out of hours availability

Different sizes and types of organisations have different challenges in supporting staff to be able to get fit tested and this illustrated a need for a variety of approaches. Examples include the need to identify a risk-assessed and safe location (or locations for community organisations), increasing the frequency of fit test sessions, drop-in fit testing, a single port of call to arrange a fit test, and the use of a checklist. Some organisations trained redeployed staff as fit testers to expand the availability of testing, and introduced large-scale fit testing to more rapidly, fit-test large numbers of staff.

Recommendation: Ensure fit testing is widely available in accessible locations to maximise ability of staff to attend

Quality of fit testing

All organisations identified a need for robust training for fit testers including competency assessment and supervised sessions to ensure safe, high quality testing for staff. Checklists were common, and one organisation described the development of a video on how to correctly undertake a fit test. Clear local policies and guidelines based on HSE guidance, both for the fit test process and for staff, were developed to ensure consistency of approach.

Organisations used a mix of qualitative and quantitative testing; this project was not designed to detect the superiority of one method over the other, and did not do so. One trust reported that quantitative fit testing enabled multiple masks to be tested at one appointment, improving both success rates and efficiency of testing.

Other measures included training of fit testers by the mask supplier, inclusion of fit testing as part of organisational mandatory training, and ensuring a supply of a variety of types of mask from suppliers. Organisations identified that the availability of multiple masks for use in one fit test, was important in ensuring success, particularly for staff who had previously had an unsuccessful fit test.

Mask alternatives

This quality improvement programme focussed on FFP3 masks. Alternatives, for example, hoods and powered respirators, are available and were used where fitting an FFP3 mask

For peer review only - http://bmjopen.bmj.com/site/about/guidelines.xhtml

was not possible (on a case by case basis). It is important to recognise that not finding mask alternatives quickly, may put staff in a position of ‘not wanting to let team down’.

HSE guidance at https://www.hse.gov.uk/respiratory-protective-equipment/fit-testing-basics.htm states that facial hair (stubble and beards) make it impossible to achieve a good seal of the respirator to the face. For staff wearing head coverings, tight fitting respirators such as FFP3 masks, the mask straps (for both fit testing and normal wear) do need to be worn in contact with the head and not the head covering. The reason for this is to lessen the chance of the straps slipping, which in turn could lead to a compromise in the face seal.

The FFP3 mask can be worn with the straps underneath the head covering. However, this should be handled sensitively - there must be a suitable environment for the wearer to don and doff the mask, and more importantly the head covering, in private.

Recommendation:

Further research into FFP3 masks and suitable alternatives, which are in line with HSE requirements, for staff who have beards or wear head coverings, is required.

Continuous measurement

Organisations highlighted the value of monitoring progress using continuous measurement - either using data collection resources provided by NHS England and NHS Improvement, or their own.

Cumulative analysis of the number of staff tested, total number of fit tests undertaken, and percentage of staff with a successful fit test enabled organisations to track progress with different types of FFP3 masks, and conduct further analysis to identify areas that required further input.

Recommendation:

- Staff experience should be discussed, with a broad range of staff representative of the demographic of staff employed by the trust, to enable mitigation of any issues quickly
- Link feedback from staff and testers, with other departments such as procurement (regarding changes in the availability of different types of masks)

Recording of results

All organisations identified the need for accurate and accessible recording of fit test outcomes, including negative results. Examples include a central database linked to health roster, registers, and certificates for staff. ESR has recently been updated to enable fit test results to be entered on staff records, which allows staff to download their results and notify other organisations if they work in more than one, or move posts. This should avoid unnecessary duplication of fit testing.

Recommendation:

- Record fit testing results at individual staff, departmental and trust level
- Continuously measure progress of fit testing using key benchmarks

For peer review only - http://bmjopen.bmj.com/site/about/guidelines.xhtml

- Ensure that all staff who are required to wear a FFP3 mask are regularly fit tested, and that there is organisational board level assurance

Redeployment

The availability of FFP3 mask alternatives such as hoods has reduced the need for redeployment. A redeployment algorithm developed by NHS England and NHS Improvement is available on the website.

https://www.england.nhs.uk/coronavirus/publication/supporting-fit-testing-steps-and-actions-to-be-taken-where-staff-may-require-the-use-of-ffp3-masks/

Recommendation:

- Ensure redeployment pathways are in place for staff who are not able to be successfully fit tested on available masks, or use alternatives.

Staff skin care

Once a member of staff has successfully been fit tested for a mask it is important to ensure that they take good care of their skin to prevent skin irritation and / or pressure ulcers. Guidance on how to help prevent facial skin damage beneath tight fitting face piece respirators (FFP 3 masks) is available.

https://www.england.nhs.uk/coronavirus/wp-content/uploads/sites/52/2020/03/C0129_Preventing_skin_damage_under_PPE_V6_29.10.20.pdf

Recommendations for fit testing

The structure of an organisation-wide fit testing programme should consider:

- **Compliance**
  - Governance structure must ensure that fit testing methodology meets with HSE standards
  - Include clear policies and guidelines
- **Fit testers**
  - Identify who they are, how many there are, the governance structure, competency assessment, their professional development and well-being
- **Staff to be fit tested**
  - Clarify the number of staff to be fit tested, their work areas, education about the fit test and support
- **Accessibility**
  - Consider the location of fit testing, number and timing of sessions, ease of booking of fit tests and the availability of urgent or short notice fit-testing
- **Equipment**
  - Quantitative and qualitative equipment required
  - Ensure consistent supply of FFP3 masks, ordering process and distribution, responsiveness to needs of the trust workforce
  - Ensure the availability of alternative PPE such as hoods

For peer review only - http://bmjopen.bmj.com/site/about/guidelines.xhtml

The following checklist was developed as part of the quality improvement programme.

The team is happy to share with resource with peers

NHS
Royal Free London
NHS Foundation Trust

Face Fit Testers
Checklist
FFP3 Disposable masks

**Fit Testers Name**		**Fit Test Method**	Qualitative
**Date**		**Assessors Name**	
**Work Location**			

	Pass	Prompt	Fail
**Understand where FFP3 masks must be worn[1]**			
Tester aware of Trust/PHE guidance on the correct PPE for task (i.e. AGP etc)			
**Can identify damage or defect in mask[2]**			
Check disposable mask for damage/defects			
**Practical - don mask and perform fit check**			
Demonstrates correct fitting of mask following IPC guidelines			
Demonstrates fit-check clearly, and instructs that it must be done every time mask is worn			
**Maintenance of testing equipment and room[3]**			
Check hood and pumps for damage/defects			
Checks nebuliser sprays			
Check room is suitable for fit test			
**Purpose fit testing and the fit test exercises**			
Can explain why we fit test staff and why we don't just do a static test			
**Limitations of qualitative fit testing[4]**			
Understands reasons to use machine test instead of taste test			
**Practical - perform a fit test**			
Checks for medical condition			
Explains test method clearly and purpose			
Sensitivity test carried out			
Understands option if user fails sensitivity test			
Ensures user performs fit check			
PPE worn if interferes with seal of RPE			
Carries out fit test exercises appropriately			
Can correct a failure in fit test (check[7])			
Only allows 1 retest on a mask model (check[7])			
Records results accurately			
Reminds retest period and reasons for retest			
**Doffing[12]**			
Can demonstrate how to safely doff and dispose of mask after use			

For peer review only

SCENARIO TEST			
	**Pass**	**Prompt**	**Fail**
**Understands problems that could prevent a successful fit test**[8]			
Identifies issues that might prevent person tasting solution			
Identifies issues with example images and suggests appropriate corrections			
**Recognise a poorly fitting FFP3 mask**			
Tester able to recognise poorly fitting mask (example images)			

[1] **Selection of adequate and suitable RPE.** Within healthcare setting the RPE is selected by IPC/PHE and fit testers would not be involved with this

[2] **Examination of RPE and the ability to identify poorly maintained facepieces** – this is for disposable only hence requirement to be able to check for defects. 3-5 example masks given with defects in some, such as missing valve, damaged seal etc.

[3] **Awareness of external factors that may affect the fit of the facepiece or the fit test result** – adjusted for QLFT rather than QNFT

[4] **The differences between, and the appropriate use of, QNFT and QLFT methods; capabilities and limitations of the fit test equipment** - adjusted to only reflect QLFT

[5] **Preparation of facepieces for fit testing** – REMOVED for QLFT

[6] **how to carry out diagnostic checks on the facepiece and the fit test equipment** – removed for QLFT

[7] When the first test is successful the knowledge should be checked by questioning

[8] Will give images of potential issues (holding a coffee, facial hair, headgear, PPE, straps worn incorrectly) tester to identify and explain how to correct

Facial Hair around the seal of the mask
Reminder to staff that they should be clean shaven when wearing RPE
Facial markings/piercing around the seal
Clothing or accessories (headscarf, glasses) that interfere with seal
Straps in wrong position

[9] **Interpretation of fit test results** – REMOVED for QLFT as the result is binary (reason for failure is already covered

[10] **Understanding of the differences between fit factor, workplace protection factor (WPF),† assigned protection factor and nominal protection factor (NPF)‡** - REMOVED N/A for NHS fit testers. RPE is decided at a national/hospital level, knowledge not required to be retained. Cover in training but this is not useful knowledge

[11] **HSE ACOPs and guidance that deal with fit testing of RPE.** - To be referred to in training, tested in the questions above.

[12] Not part of the HSE but felt it is necessary to add to NHS testing

For peer review only - http://bmjopen.bmj.com/site/about/guidelines.xhtml

Fit testing suggested reading

NHS England and NHS Improvement (2020) FFP3 respirators and other facial personal protective equipment (PPE). Available at: https://www.england.nhs.uk/coronavirus/secondary-care/infection-control/ppe/ffp3/ (Accessed 4 November 2020).

NHS England and NHS Improvement (2020) Fit testing algorithm. Available at: https://www.england.nhs.uk/coronavirus/publication/supporting-fit-testing-steps-and-actions-to-be-taken-where-staff-may-require-the-use-of-ffp3-masks/ (Accessed 4 November 2020).

NHS England and NHS Improvement (2020) Helping to prevent skin damage beneath tight fitting face respirators. Available at: https://www.england.nhs.uk/coronavirus/wp-content/uploads/sites/52/2020/03/C0129_Preventing_skin_damage_under_PPE_V6_29.10.20.pdf (Accessed 17 November 2020).

Public Health England, (2020a) Understanding the data: the impact of Covid 19 on BAME communities. Available at: https://assets.publishing.service.gov.uk/government/uploads/system/uploads/attachment_data/file/892376/COVID_stakeholder_engagement_synthesis_beyond_the_data.pdf [Accessed 19 October 2020]

Public Health England, (2020b) Disparities on the risk and outcomes of Covid 19. Available at: https://assets.publishing.service.gov.uk/government/uploads/system/uploads/attachment_data/file/908434/Disparities_in_the_risk_and_outcomes_of_COVID_August_2020_update.pdf_[Accessed 19 October 2020]

HSE guidance

HSE Guidance on respiratory protective equipment at work HSG53 https://www.hse.gov.uk/pubns/books/hsg53.htm

HSE Guidance on respiratory protective equipment (RPE) fit testing INDG479 https://www.hse.gov.uk/pubns/indg479.pdf

HSE website page on fit testing https://www.hse.gov.uk/respiratory-protective-equipment/fit-testing-basics.htm

HSE guidance on fit testing during COVID-19 https://www.hse.gov.uk/coronavirus/ppe-face-masks/face-mask-ppe-rpe.htm

British Industry Safety Federation Fit2fit companion guides https://www.bsif.co.uk/fit2fit-companions-released/

For peer review only - http://bmjopen.bmj.com/site/about/guidelines.xhtml

Appendix B

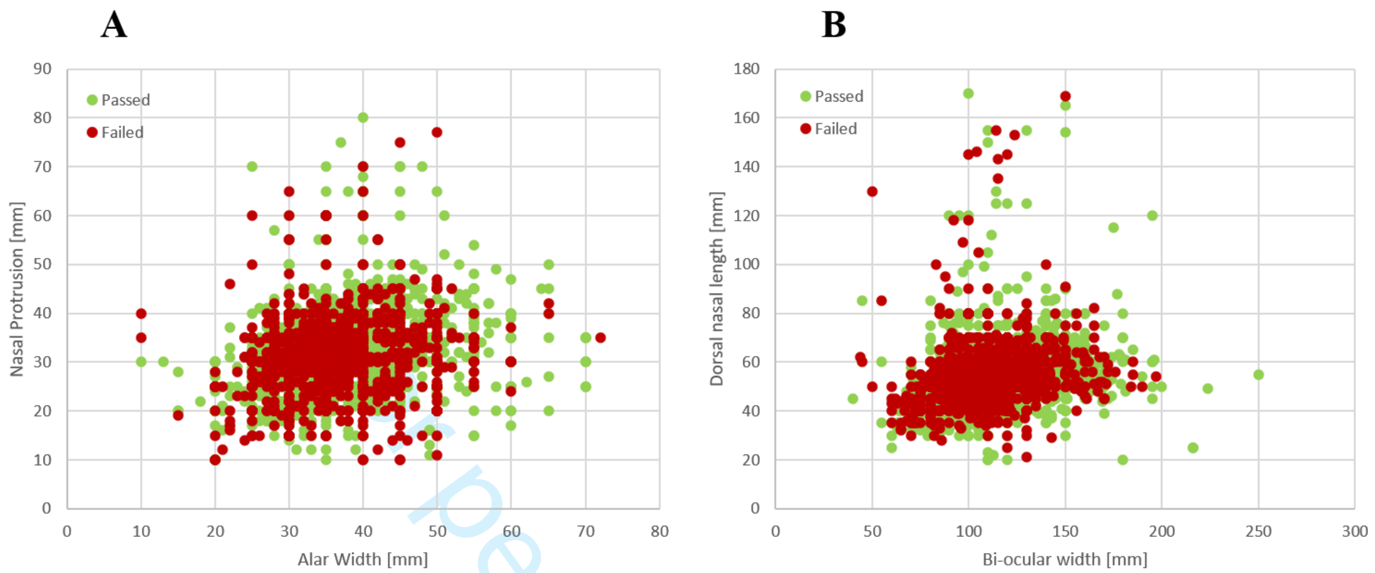

Categorical scatter plots of alar width [mm] versus nasal protrusion [mm] (A) and bi-ocular width [mm] versus dorsal nasal length [mm] (B) with respect to failed and passed fit tests at attempt 1, represented by red and green markers, respectively.

For peer review only

Revised Standards for Quality Improvement Reporting Excellence (SQUIRE 2.0)
September 15, 2015

Text Section and Item Name	Section or Item Description
**Notes to authors**	• The SQUIRE guidelines provide a framework for reporting new knowledge about how to improve healthcare<br><br>• The SQUIRE guidelines are intended for reports that describe system level work to improve the quality, safety, and value of healthcare, and used methods to establish that observed outcomes were due to the intervention(s).<br><br>• A range of approaches exists for improving healthcare. SQUIRE may be adapted for reporting any of these.<br><br>• Authors should consider every SQUIRE item, but it may be inappropriate or unnecessary to include every SQUIRE element in a particular manuscript.<br><br>• The SQUIRE Glossary contains definitions of many of the key words in SQUIRE.<br><br>• The Explanation and Elaboration document provides specific examples of well-written SQUIRE items, and an in-depth explanation of each item.<br><br>• Please cite SQUIRE when it is used to write a manuscript.
**Title and Abstract**	
**1. Title**	**Page 1, Line 5**<br>Factors affecting successful fit testing of Respiratory Protective Equipment during COVID-19: A National Quality Improvement Initiative.

For peer review only

**2. Abstract**	**Page 2, Line 31**<br><br>**Introduction** Respiratory protective equipment has been a critical means to protect healthcare workers from COVID-19 infection. These devices are typically single use Filtering Facepiece Respirators, which are affixed to the face to prevent airborne transmission of the virus. There have been several reports of fitting issues within particular sub-groups of healthcare workers, although the factors affecting successful fitting of masks are still largely unknown.<br><br>**Objective** To evaluate factors affecting respirator fitting outcomes within a national health service during the COVID-19 pandemic.<br><br>**Methods** We conducted a secondary analysis of a national database involving fit testing outcomes collated between July to August 2020. Key demographics including age, gender, ethnicity and face measurements were compared to fitting outcome in a variety of respirator mask designs. A mixed-effects logistic regression model was used to determine the Odds Ratio for factors which affected fit testing outcome.<br><br>**Results** A total of 9,592 observations from 5,544 healthcare workers working with 47 UK Hospital Centres were included in the analysis. The mixed-effects logistic regression model identified that males experienced a significantly ($p<0.05$) higher fit test success than females (OR = 1.57; 95% CI: 1.31 to 1.88). Those with non-white ethnicities demonstrated lower odds of successful mask fitting, with black (OR = 0.66; 95% CI: 0.51 to 0.86), Asian (OR = 0.60; 95% CI: 0.50 to 0.71) and mixed (OR = 0.79; 95% CI: 0.64 to 0.99) ethnic groups all being statistically significant ($p<0.05$). There were significant differences ($p<0.05$) between mask models, when controlling for sex, age and ethnicity.<br><br>**Conclusion** During the early phase of COVID-19, females and non-White ethnicities were less likely to have a successful respirator mask fitting in England, with a high degree in variability regarding mask model fitting outcomes. Due to the diverse workforce, healthcare settings require a range of masks to ensure successful fitting outcomes. Further improvements with industry representatives [illegible]
**Introduction**	*Why did you start?*
**3. Problem Description**	**Page 4, Line 94**<br><br>The use of respiratory protective equipment (RPE) is vital in the prevention of nosocomial viral transmission for conditions such as influenza, SARS and SARS-CoV-2 (COVID-19).

For peer review only

**4. Available knowledge**	**Page 4, line 104**<br><br>There are many factors which may influence successful RPE fitting [11]. These include the relationship between facial dimensions and RPE shape and the materials used within the mask which include both compliant and non-compliant designs [12]. Indeed, facial anthropometrics vary significantly across HCWs, between genders, age, ethnicities, occupational groups and on an individual basis. These differences will influence the performance of RPE and there is already some evidence, albeit contradictory, that overall protection varies with gender-based differences in facial dimension [13-16]. These findings are of clear relevance for the design and manufacture of respirators, which need to match the characteristics of HCWs who are predominantly female and often from ethnic minority communities [17]

For peer review only

5. **Rationale**	**Page 4, line 118**<br><br>In the UK, the National Health Service (NHS) has purchased a variety of FFP3 RPE devices to protect frontline HCWs in response to SARS-CoV-2 (COVID-19) as part of their pandemic preparedness stockpile. However, it is not known how key demographics of healthcare workers effects fitting outcomes of FFP3 masks.
6. **Specific aims**	**Page 4, line 124**<br>Aim is to analyze data from a quality improvement initiative to answer the following research questions:<br>1. Do intrinsic factors, namely, gender identity, ethnic background, age and BMI affect the success rates of FFP3 mask fitting?<br>2. Is there a variation in performance of specific mask models in terms of fit test success over the number of attempts?<br>3. Do specific mask models match subject classification in terms of gender and ethnic group?<br>4. Do individual facial measurement parameters reflect the likelihood of [illegible]
**Methods**	*What did you do?*
7. **Context**	**Page 5, Line 133**<br>This retrospective evaluation study, involving National Health Service (NHS) hospitals in England, used data detailing fit test outcomes in all healthcare workers in the early stages of the COVID-19 pandemic.
8. **Intervention(s)**	**Page 5, line 135**<br>Fit testing of Filtering Facepiece Respirators (FFP3 masks) on a cohort of healthcare workers in England, working in the national health service.
**9. Study of the Intervention(s)**	**Page 5, line 144**<br>The outcomes is the successful fitting of the FFP3 mask from fit testing clinics in the NHS.

For peer review only

**10. Measures**	**Page 5, line 149**<br>Data included the type and make of RPE models used and success or failure of fitting according to two standardised and prescribed methods from the Health and Safety Executive, namely the qualitative fit test (QLFT) or the quantitative fit test (QNFT) [18 19]. This was conducted with a ToolKit provided (e-Appendix) to each hospital trust, through which details of demographics were collected, including gender, ethnic background, age and BMI. In the latter case, BMIs were categories using the World Health Organization (WHO) classifications as underweight (<18.5 kg/m^2), optimal (18.5-25 kg/m^2), overweight (25-30 kg/m^2) and obese (>30 kg/m^2) [20]. In addition, five facial anthropometrical measurements were estimated with a paper-based ruler, namely, facial length, nasal length and protrusion, alar and bi-ocular width.
**11. Analysis**	**Page 6, line 186**<br>Cleaned data was imported into Stata (Stata 16.0, StataCorp LLC, USA), where a basic model assessment was performed using the Bayesian information criterion (BIC). The model with the lowest BIC value was selected incorporating the mask model, gender, ethnicity and age. Following this evaluation, a mixed-effects logistic regression model was used to determine the ORs and their 95% confidence intervals (95% CI) for factors which affected fit testing outcome using a collated data set across each of the five fit test attempts. Analysis was conducted while adjusting for multiple risk factors and potential confounders. This approach accounted for nested data (as several, different masks were fitted to the same person) and for within-subject correlation by means of a random effect for subject.
**12. Ethical Considerations**	**Page 17, line 442**<br>Institutional ethics was approved to retrospectively analyse an anonymised version of the data set (ERGO-65166). Data was confidentially shared with the research group at the University of Southampton (UoS) and stored in accordance with the Data Protection Act 2018.
**Results**	*What did you find?*
**13. Results**	**Page 7, line 201**<br>A total of 9,592 observations from 5,512 healthcare workers were included in the regression model. Most of the individuals, approximately 78% of the total for all attempts, were aged between 24 and 54 years of age. Those that were younger (18-24 years) represented 9% of the cohort, whereas those in the older categories (up to 74 years of age) represented 13% of the participants.<br>The mixed-effects logistic regression model identified that males experienced a significantly ($p<0.05$) higher fit test success than females (OR = 1.57; 95% CI: 1.31 to 1.88). Those with non-white ethnicities demonstrated lower odds of successful mask fitting, with black (OR = 0.66; 95% CI: 0.51 to 0.86), Asian (OR = 0.60; 95% CI: 0.50 to 0.71) and mixed (OR = 0.79; 95% CI: 0.64 to 0.99) ethnic groups all being statistically significant ($p<0.05$). There were significant differences ($p<0.05$) between mask models, when controlling for sex, age and ethnicity.

For peer review only

**Discussion**	*What does it mean?*
**14. Summary**	**Page 14, line 309**<br>During the early phase of COVID-19, females and non-White ethnicities were less likely to have a successful respirator mask fitting in England, with a high degree in variability regarding mask model fitting outcomes.

For peer review only

**15. Interpretation**	**Page 14, line 336**<br>Due to the diverse workforce, healthcare settings require a range of masks to ensure successful fitting outcomes. Further improvements with industry representatives and standards committees is required to ensure mask designs are fit for purpose.
**16. Limitations**	**Page 15, line 350**<br>This secondary analysis of quality improvement data was limited by the nature in which individual hospital trust collected and documented fit testing. Indeed, a small proportion of data was omitted during the data cleaning process in order to conduct robust analysis. The results of this retrospective analysis are only reflective of the time period of investigation (July-August 2020), with improvements to mask designs and fitting processes evident during the latter stages of the pandemic. The data presented in the present study is also a product of the international demands on PPE supply chain 3, with a diverse range of masks included in the data set. As a result, RPE in different English hospitals was variable and procurement dependent. In addition, implementing a comprehensive fit-testing program is a financial and logistical challenge 43, limiting the feasibility to test all HCWs on different FFP3 mask models.
**17. Conclusions**	**Page 16, line 389**<br>This secondary analysis of fit testing outcomes derived from a multi-centre data set revealed a range of FFP3 mask models were procured in the early stages (July and August 2020) of the pandemic to protect HCWs involved in managing COVID-19 patients. The data identified distinct trends in fit testing outcomes related to both gender and ethnicity. Indeed, white males were the most likely to yield a successful fit test outcome and ethnic minority females the least likely. In many cases, repeat fit testing with different respirator models was required to ensure individual staff had a correctly fitting device. Further research is needed to provide improvements in the fit testing approach resulting in a more intelligent selection process. In addition, collaborative working with industry and standards representatives is required to optimise RPE designs to accommodate a wide range of face shapes incorporating different genders and ethnicities. In addition, mask designs and material interfaces need to support the protection of skin health for those who require devices for prolonged periods.
**Other information**	
**18. Funding**	**Page 16, line 406**<br>The secondary analysis of data was supported by UK Research and Innovation (UKRI) as part of a funded project "A Bio-Engineering approach for the SAFE design and fitting of Respiratory Protective Equipment (BE-SAFE RPE)" (Ref EP/V045563/1).

For peer review only

Table 2. Glossary of key terms used in SQUIRE 2.0. This Glossary provides the intended meaning of selected words and phrases as they are used in the SQUIRE 2.0 Guidelines. They may, and often do, have different meanings in other disciplines, situations, and settings .

Assumptions
Reasons for choosing the activities and tools used to bring about changes in healthcare services at the system level.

Context
Physical and sociocultural makeup of the local environment (for example, external environmental factors, organizational dynamics, collaboration, resources, leadership, and the like), and the interpretation of these factors ("sense-making") by the healthcare delivery professionals, patients, and caregivers that can affect the effectiveness and generalizab ility of intervention(s).

Ethical aspects
The value of system-level initiatives relative to their potential for harm, burden, and cost to the stakeholders. Potential harms particularly associated with efforts to improve the quality, safety, and value of healthcare services include opportunity costs, invasion of privacy, and staff distress resulting from disclosure of poor performance.

Generalizability
The likelihood that the interve ntio n(s) in a particular report would produce similar results in other settings, situations, or environments (also referred to as external validity).

Healthcare improvement
Any systematic effort intended to raise the quality, safety, and value of healthcare services, usually done at the system level. We encourage the use of this phrase rather than "quality improvement," which often refers to more narrowly defined approaches.

Inferences
The meaning of findings or data, as interpreted by the stakeholders in healthcare services – improvers, healthcare delivery professionals, and/or patients and families

Initiative
A broad term that can refer to organization-wide programs, narrowly focused projects, or the details of specific interventions (for example, planning, execution, and assessment)

Internal validity
Demonstrable, credible evidence for efficacy (meaningful impact or change) resulting from introduction of a specific intervention into a particular healthcare system.

Intervention(s)
The specific activities and tools introduced into a healthcare system with the aim of changing its performance for the better. Complete description of an intervention includes its inputs, internal activities, and outputs (in the form of a logic model, for example), and the mechanism(s) by which these components are expected to produce changes in a ystem's performance.

Opportunity costs

For peer review only

Loss of the ability to perform other tasks or meet other responsibilities resulting from the diversion of resources needed to introduce, test, or sustain a particular improve me nt initiative

Problem

Meaningful disruption, failure, inadequacy, distress, confusion or other dysfunction in a healthcare service delivery system that adversely affects patients, staff, or the system as a whole, or that prevents care from reaching its full potential

Process

The routines and other activities through which healthcare services are delivered

Rationale

Explanation of why particular interventio n(s) were chosen and why it was expected to work, be sustainable, and be replicable elsewhere.

Systems

The interrelated structures, people, processes, and activities that together create healthcare services for and with individual patients and populations. For example, systems exist from the personal self-care system of a patient, to the individual provider-patient dyad system, to the microsystem, to the macrosystem, and all the way to the market/social/insurance system. These levels are nested within each other.

Theory or theories

Any "reason-giving" account that asserts causal relationships between variables (causal theory) or that makes sense of an otherwise obscure process or situation (explanatory theory). Theories come in many forms, and serve different purposes in the phases of improve me nt work. It is important to be explicit and well-founded about any informal and formal theory (or theories) that are used.

For peer review only
